# Exploring the impact of cross-cultural training on cultural competence and cultural intelligence: a narrative systematic literature review

**DOI:** 10.3389/fpsyg.2025.1511788

**Published:** 2025-04-07

**Authors:** Deniz Urgun, Johanna Seidel, Eleni Vangeli, Mario Borges, Rita F. de Oliveira

**Affiliations:** ^1^School of Applied Sciences, London South Bank University, London, United Kingdom; ^2^Department of Health and Social Psychology, German Sport University Cologne, Cologne, Germany

**Keywords:** diversity, acculturation, intercultural, expatriate, migrant, coach education, professional development

## Abstract

**Introduction:**

Cross-cultural training programs are widely used to enhance cultural competence and cultural intelligence (CQ) across various professional fields. This narrative systematic literature review examines training strategies from various fields to identify the most effective approaches for application in sports. It addresses two key research questions: (1) What training components have been used in the delivery of these training programs? (2) What is their effectiveness in improving cultural intelligence and cultural competence?

**Methods:**

A systematic search was conducted to identify qualitative and quantitative studies published between 2000 and 2023. A total of 27 articles met the inclusion criteria. These articles describe the type of training program delivered, the activities performed, and their outcomes on participants’ cultural competence and/or cultural intelligence. Programs were categorized on delivery methods (didactic, experiential, or mixed) and information was extracted on training content, participants, duration, and timing.

**Results:**

Most programs used mixed delivery methods that combined lectures, educational tasks and experiential activities. They showed positive, though not always statistically significant, impact on participants’ cultural competence and/or cultural intelligence.

**Discussion:**

In sports, tailored programs that address culture- and sport-relevant knowledge, skills and communication may help coaches navigate cultural differences.

## Introduction

Sports migration and globalization increasingly mean that sports coaches and athletes work in culturally diverse contexts, with an expectation of effective performance and interactions. Coaches often choose to migrate to develop their sport or their career (Borges et al., 2015, 2020, 2023, 2024) but whether abroad or within their home country, cross-cultural interactions can pose challenges. Challenges for sports coaches can be due to cultural differences, such as those between foreign and domestic players and coaches (Khomutova, 2015; Sain et al., 2022), communication and language barriers (Borges et al., 2022a), and the need to understand new cultural norms and values (Schinke et al., 2013; Borges et al., 2015). Additionally, challenges may arise from a lack of familiarity with the new environment (Ryba et al., 2013) and from understanding the unique needs of the athletes they work with (Duchesne et al., 2011). Therefore, cross-cultural training is a useful educational tool aimed at promoting intercultural learning to enhance individuals’ cultural awareness and improve their cultural competence to manage cultural differences (Borges et al., 2022a, 2022b, 2023; Chen, 2015).

Despite various conceptualizations of cultural competence, there is a consensus that it refers to the ability to function effectively across different cultures (Whaley and Davis, 2007). In their review of cultural competence models, Leung et al. (2014) identified more than 30 models and more than 300 related constructs, which generally adopt an individual-differences approach (Sandberg, 2000). By taking this approach, cultural competence was conceptualized as a set of personal characteristics, which are summarized into three content domains of (a) intercultural traits, (b) intercultural capabilities, and (c) intercultural attitudes and worldviews. Cultural competence models grounded in personality mostly focus on intercultural traits, while models drawing on the intelligence literature focus on intercultural capabilities, including cultural intelligence (Leung et al., 2014). Regarding cultural competence in the sports context, Burden and Lambie (2011) developed the Sociocultural Competencies for Sports Coaches (SCSC), by adapting the competencies model from Sue et al. (1992). These sociocultural competencies focus on attitudes/beliefs and skills and specify knowledge as an important domain (but not traits). Specifically, it comprises three competencies: (a) coaches’ self-awareness of personal biases and beliefs, (b) coaching skills and strategies, and (c) knowledge of athletes’ worldviews.

One of the most prominent models is the Cultural Intelligence Model (Earley and Ang, 2003) which has shown to predict various outcomes of interest (Leung et al., 2014; Matsumoto and Hwang, 2013). Cultural intelligence (CQ) refers to a person’s capability to function effectively in multicultural settings, reflected in a set of capabilities (Ang and Van Dyne, 2015). The conceptualization of cultural intelligence draws upon the Sternberg and Detterman’s (1986) multi-loci theory of intelligence, consisting of four dimensions: motivational, metacognitive, cognitive, and behavioral, along with their associated sub-dimensions (Earley and Ang, 2003). Metacognitive CQ pertains to the cognitive processes and mental capacity to acquire cultural knowledge, whereas cognitive CQ refers to knowledge structures concerning cultures and cultural distinctions. Motivational CQ pertains to the ability to direct and maintain energy to function and perform effectively in intercultural contexts while behavioral CQ is the capacity to modify behaviors (verbal and nonverbal) to function appropriately in various cultures. For instance, an individual possessing a higher behavioral CQ might adapt one’s verbal and non-verbal behaviors to suit various cultures, while an individual with a higher metacognitive CQ is more likely to formulate action plans before engaging in cultural interactions.

Cultural intelligence is a specific form of intelligence, and it distinguishes itself from other prominent approaches to cultural competence (Van Dyne et al., 2017). For example, cultural competence models rarely consider all dimensions and factors simultaneously, whereas the cultural intelligence model is comprehensive (Ang et al., 2015). Moreover, cultural competence models focus on a variety of narrower personality dimensions (e.g., self-awareness, cognitive flexibility), whereas cultural intelligence model focusses on broader intercultural capabilities (Ang et al., 2015). Prior studies have extensively examined the concepts of cultural competence and cultural intelligence, as well as the outcomes of cross-cultural training across multiple domains. Nevertheless, there is a paucity of research conducted in sports settings. A recent study by Borges et al. (2022b) investigated the cross-cultural training needs of 209 football coaches and addressed the concept of cultural intelligence in sports. They found that coaches with international experience rated their cultural intelligence (CQ) higher than those without such experience. In addition, the study found that coaches preferred training focused on cross-cultural communication skills, delivered through practice-based methods with in-person attendance, and conducted before moving to a foreign country. While this study offers initial insights into cross-cultural training for sports, further research is needed to determine the specific training elements required for coaches across various sports.

To our knowledge, no other studies have explored sport-specific cross-cultural training, as such programs have yet to be developed and implemented. This study aimed to gather and synthetize information from training programs in different fields with a view to later transferring the most effective strategies to the sports field. We expect cultural training programs to have a positive and significant impact on participants’ cultural competence and cultural intelligence (CQ), with varying effects across different dimensions depending on the type of program delivery.

## Research questions

The following research questions were formulated using the PICO framework to specify the population (adult participants), the intervention was cultural training programs, the comparator included pre-post designs, no training, or any alternative training, and the outcomes measured (quantitative or qualitative) effectiveness in terms of cultural intelligence and/or cultural competence either qualitative.

*RQ1*: What training components (i.e., content, delivery type, duration) have been used in the delivery of cross-cultural training programs aimed at improving the cultural intelligence and/or cultural competence of participants across different multicultural settings?

*RQ2*: What is the effectiveness of cross-cultural training programs in improving the cultural intelligence and cultural competence of participants across different multicultural settings, as assessed through quantitative and/or qualitative study designs?

## Method

The systematic review was carried out in accordance with the PRISMA 2020 statement (Page et al., 2021) and used the PRISMA checklist protocol (Rethlefsen et al., 2021) which enables researchers to identify critical areas of focus for research and facilitates the procedure for those who conduct the research.

### Eligibility criteria

For the review, we were interested in studies that used a cross-cultural training program and measured its effectiveness in terms of cultural competence and cultural intelligence.

### Information sources

The literature search was conducted using the EBSCOhost interface which searched the following databases: Academic Search Complete, Education Research Complete, MEDLINE, ERIC, APA PsycInfo, Business Source Premier, CINAHL Complete, SocINDEX with Full Text, Hospitality & Tourism Complete, Teacher Reference Center, SPORTDiscuss with Full Text. A manual search of key articles’ reference lists was also conducted for papers that were either highly cited or papers that became clear through the research as being highly relevant.

### Search strategy

To ensure a comprehensive but specific search for eligible papers, the search terms were first developed and tested by the researchers. The final search terms used were: (“cross-cultural training” or “cct” or “cross-cultural program” or “cross-cultural education”) AND (“cultural intelligence” or “cultural competence”). The limiters developed in this review were: “peer-reviewed,” “published date (2000–2023),” and the language selected as “English.” We included studies from 2000 to 2023 to ensure our review reflects current evidence and the latest developments in training programs (Moher and Tsertsvadze, 2006). The search terms and limiters were used for all databases using the EBSCOhost interface. See Table 1 for inclusion and exclusion criteria.

**Table 1:** Inclusion and exclusion criteria used for the study.

Feature	Inclusion	Exclusion
Language	Published in the English language	Papers written in a language other than English
Date of publication	Papers published between 2000–2023	Papers published prior to 2000
Type of studies	Peer-reviewed empirical papers Papers with the keyword “cultural intelligence” or “cross-cultural training” in the abstract Papers included the within subject and/or between subject design, or both Papers included qualitative and/or quantitative research methods Papers reported cross-cultural training program delivery methods (i.e., content, delivery type, duration, and participants) Papers reported the impact of cross-cultural training programs on CQ and/or cultural competence	Extended abstracts, posters, books, theses, chapters, unpublished material, or short papers about research that is still going on Conference papers

### Selection process

**Figure 1:**
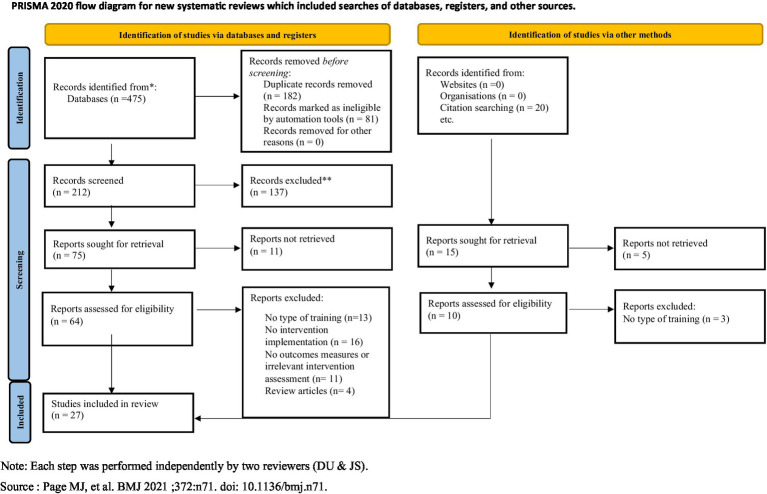
PRISMA flow chart of the study selection process.

The initial search conducted by DU resulted in 475 papers. After the removal of duplicates and records automatically marked as eligible by automation tools, DU and JS independently screened all study titles and abstracts for inclusion and any disagreements were discussed and resolved by referring back to the eligibility criteria. DU and JS then reviewed all the full-text papers. Of the 64 papers, six were discussed between DU and JS and agreed, and 8 were discussed with RO or MB and agreed. Only three papers were discussed with RO/MB, which changed the decision taken previously. This process resulted in 20 papers, with seven additional papers added through citations resulting in the final inclusion of 27 papers (Figure 1). The full text of excluded papers is included in the Supplementary material.

### Data collection process

Details of the 27 papers were extracted and compiled into a table (DU and JS). Any uncertainties and disagreements regarding the variables during data extraction were resolved through discussions between authors. As an example, six papers were excluded because they contained insufficient information concerning the type and implementation of the training. Authors were not contacted to provide further information about the training programs.

### Data items

For each paper, data was collected about the components of the cross-cultural training program (delivery method, participants, content, duration) and its outcomes on cultural intelligence and cultural competence, as well as study design.

### Quality assessment

A quality assessment of papers was not performed as the focus was on synthesizing findings related to the types and impacts of training programs, rather than on evaluating the methodological quality of the individual studies.

### Synthesis method

A narrative synthesis was used because it is the most appropriate way to discuss the different components of training delivery as per our main research question.

## Results

The reviewed studies delivered training across a variety of participant groups including students at different education levels and settings (*n* = 21), working professionals (*n* = 3), psychiatry nurse residents (*n* = 1), military and government members (*n* = 1), and mental health professionals (*n* = 1). Most training was offered in person, often supplemented by online resources, with only one program delivered entirely online (see Kirste and Holtbrügge, 2019). The duration of these programs ranged from a full-day workshop (Hiller and Woźniak, 2009) to a six-month course (Presbitero and Toledano, 2018). Additionally, five programs required participants to spend time abroad lasting from 1 week to 4 months, with four of these offering pre-departure training (Alexander et al., 2021; Alexander et al., 2022; Eisenberg et al., 2013; Engle and Crowne, 2014). See Supplementary Table 1 for detailed information.

*RQ1*: What training components (i.e., content, delivery type, duration) have been used in the delivery of cross-cultural training programs aimed at improving the cultural intelligence and/or cultural competence of participants across different multicultural settings?

In response to this research question, we categorized the studies into three delivery types: (1) didactic, (2) experiential, and (3) mixed delivery methods (See Supplementary Table 2 for detailed summaries of each program’s content and components).

### Didactic delivery

Seven studies employed didactic methods, incorporating various activities: courses, group discussions, briefings, as well as self-directed and group-directed tasks relevant to the cultural topics (Harris et al., 2008; Pandey, 2012; Rehg et al., 2012; Smith and Bahr, 2014; Spitzer, 2015; Ramsey and Lorenz, 2016; Kirste and Holtbrügge, 2019). The primary focus of these courses was on culture-general and culture-specific knowledge. For instance, Smith and Bahr (2014) offered an overview of cultural competence, discussing its definitions and presenting cultural characteristics of the Hispanic population including aspects like second-language acquisition and parental involvement (i.e., culture-general and specific knowledge). Likewise, Harris et al. (2008) provided psychiatry residents with general cultural knowledge as well as culturally sensitive techniques for interviewing, diagnosing, and reviewing patients tailored to their practice settings.

Group activities mostly consisted of discussions, briefings, and presentations while individual activities included tasks such as self-reflection essays, case studies, and readings. For example, Spitzer’s (2015) program asked students to create surveys and compare responses from first- or second-generation immigrants to typical acculturation patterns described in the literature. In Pandey’s (2012) workshop, participants analyzed video clips from popular films such as “Outsourced” and “My Big Fat Greek Wedding.” Of the seven programs using a didactic delivery, only one relied entirely on online resources, with participants completing four training units and exercises over 2 weeks (Kirste and Holtbrügge, 2019).

### Experiential delivery

Seven studies used experiential delivery including activities such as simulation games (Bücker and Korzilius, 2015; Rahayu and Arga, 2019) and cultural immersion (Wood and St. Peters, 2014; MacNab, 2012; Alexandra, 2018a, 2018b) followed by group discussions. For example, Wood and St. Peters (2014) developed cross-cultural study tours, immersing participants in unfamiliar cultural environments through visits to organizations and community engagement. MacNab (2012) and Alexandra (2018a, 2018b) used self-selected cultural immersion experiences, combined with group reflection program as part of the *7-stage Experiential CQ Education* program. Majda et al. (2021) introduced a set of six experiential activities, such as role-playing and simulation exercises to improve nursing students’ cross-cultural communication skills and CQ. Overall, experiential programs relied on interactive methods like simulations and immersion to foster cultural awareness and practical skills within multicultural settings.

### Mixed delivery

Thirteen studies used a mixed approach combining didactic and experiential methods. These programs typically combined courses, educational tasks and experiential activities. Courses addressed cultural concepts, such as CQ and leadership (Azevedo and Shane, 2019), the functioning and operation of cultures (Presbitero and Toledano, 2018), cultural dimensions (Eisenberg et al., 2013), and cultural competence (Kratzke and Bertolo, 2013). Tasks included film assignments (Engle and Crowne, 2014; Azevedo and Shane, 2019), presentations (Azevedo and Shane, 2019; Dunlap and Mapp, 2017), and journaling on cultural experiences (Alexander et al., 2021; Alexander et al., 2022; Azevedo and Shane, 2019). The experiential components ranged from role playing to simulations and cross-cultural conversations (Kurpis and Hunter, 2017; Young et al., 2018).

Some training programs (*n* = 4) used preparatory activities related to field trips abroad (Eisenberg et al., 2013; Alexander et al., 2021; Alexander et al., 2022; Engle and Crowne, 2014). For instance, Engle and Crowne’s (2014) program included preparatory readings, film tasks, and cultural case studies. After their international experiences, participants took part in debriefings and were encouraged to submit cultural case studies for future students. Similarly, Eisenberg et al. (2013) used cultural preparation before study abroad programs, leading to significant gains in overall CQ.

Role-playing activities, such as the BaFa BaFa game, were used to simulate cross-cultural interactions (Hiller and Woźniak, 2009; Fischer, 2011; Kratzke and Bertolo, 2013). These activities engaged participants in cultural role assignments, encouraging them to reflect on their reactions and the behaviors of others in foreign cultures.

Self-reflective essays were also a key component in several programs (Kratzke and Bertolo, 2013; Dunlap and Mapp, 2017; Alexander et al., 2021; Alexander et al., 2022; Azevedo and Shane, 2019), aiming to improve cultural self-awareness and deepen the understanding of cross-cultural interactions. The goal of these training programs was to help participants understand their own behaviors and the reactions of individuals from different cultures. For example, Azevedo and Shane (2019) required trainees to interview a manager facing a cultural challenge and write a reflective essay, and Young et al. (2018) asked trainees to interact with refugees from diverse cultures and reflect on their experiences.

*RQ2*: What is the effectiveness of cross-cultural training programs in improving the cultural intelligence and cultural competence of participants across different multicultural settings, as assessed through quantitative and/or qualitative study designs?

This section includes a synthesis of qualitative and quantitative results reported in the reviewed studies. Overall, the findings point to positive outcomes for participants engaging in cross-cultural training programs, though not all effects were statistically significant.

### Qualitative outcomes

Eight studies (four using qualitative methods only) reported improvements in participants’ cross-cultural competence (Hiller and Woźniak, 2009; Kratzke and Bertolo, 2013; Pandey, 2012; Spitzer, 2015; Smith and Bahr, 2014; Fakhreldin et al., 2021; Kurpis and Hunter, 2017; MacNab, 2012; see Supplementary Table 3). Participants described benefits as gaining greater cross-cultural communication skills (Pandey, 2012; Kratzke and Bertolo, 2013), cultural awareness, culture-general knowledge, and cultural competence (Pandey, 2012; Spitzer, 2015). These benefits may have been associated with the specific activities in which they were involved. For example, readings and case studies program led to a better understanding of culture-related components such as cultural competence (Pandey, 2012; Spitzer, 2015), while role-playing and simulation exercises were particularly effective in enhancing communication (Kratzke and Bertolo, 2013), behavioral flexibility, empathy, and confidence in interacting with different cultural groups (Hiller and Woźniak, 2009).

### Quantitative outcomes

Nineteen studies quantitatively assessed outcomes, generally reporting positive effects on CQ and cultural competence, though some findings were not statistically significant (see Supplementary Table 4).

For instance, four of six programs using didactic methods showed improvements in cultural competence (Harris et al., 2008) and in cultural intelligence and/or its dimensions (Rehg et al., 2012; Smith and Bahr, 2014; Ramsey and Lorenz, 2016). Ramsey and Lorenz (2016) found that a cross-cultural management course, which included cultural concepts and video clips as part of the educational tasks, had a significant and positive impact on the CQ scores of the experimental group. Similarly, Rehg et al. (2012) found that a cross-cultural awareness course significantly improved participants’ cognitive and behavioral dimensions of CQ (Class 2), with smaller gains in motivational CQ, likely due to the course’s focus on culture-specific knowledge and the influence of cultural backgrounds on behavior. Harris et al. (2008) also found lasting improvements in cultural competence which were maintained at a 9-month follow-up. In contrast, one study found no positive effects of an online-only CQ training program on students’ CQ levels (Kirste and Holtbrügge, 2019), raising questions regarding the effectiveness of online-only education compared to in-person approaches.

Experiential methods consistently showed improvements to cultural competence and cultural intelligence and/or its dimensions (MacNab, 2012; Wood and St. Peters, 2014; Alexandra, 2018a, 2018b; Rahayu and Arga, 2019; Bücker and Korzilius, 2015; Majda et al., 2021). For example, participating in the 7-stage CQ education program led to improvements across all domains of cultural intelligence among participants (Alexandra, 2018a, 2018b; MacNab, 2012). However, some studies reported varied outcomes across CQ dimensions such as significant gains in cognitive, metacognitive, and motivational CQ but no change in behavioral CQ, despite the practical cross-cultural activities (Wood and St. Peters, 2014). Similarly, the Ecotonos program was effective in developing the metacognitive, behavioral, and motivational CQ, but less effective in changing cognitive CQ (Bücker and Korzilius, 2015). Significant improvements in CQ did not translate into increased cultural competence in Majda and colleagues’ program (2021).

Nine of ten programs that used mixed delivery methods showed improvements in cultural intelligence and/or its dimensions (Eisenberg et al., 2013; Engle and Crowne, 2014; Dunlap and Mapp, 2017; Presbitero and Toledano, 2018; Young et al., 2018; Azevedo and Shane, 2019; Alexander et al., 2021; Alexander et al., 2022; Fakhreldin et al., 2021). For instance, Alexander et al. (2021) combined a cultural learning course with a study abroad experience of either 3 weeks or 6 weeks. Their first study reported significant increases in all CQ dimensions, except motivational CQ, suggesting that cultural immersion did not enhance participants’ motivation. However, their 2022 study showed positive results on all CQ domains, regardless of the duration of the study abroad experience. In another study, Azevedo and Shane (2019) compared the CQ scores of students and professionals after both groups completed a similar cultural intelligence training program. While students reported significant increases in all dimensions of CQ, including a moderate increase in behavioral CQ, professionals only showed significant improvement in the cognitive dimension, with no significant changes in other areas. The differences in outcomes may stem from program differences in activities or learning preferences between groups.

Engle and Crowne (2014) showed that short-term international experiences were effective in improving CQ, while both Dunlap and Mapp (2017) and Eisenberg et al. (2013) found that pre-departure courses focusing on the target country’s culture enhanced overall CQ. However, they also found poor results in motivational and behavioral CQ despite the experiential nature of the program.

Kurpis and Hunter (2017) found that international students scored higher in cognitive CQ than domestic students, despite receiving no formal training. Both international and domestic students perceived benefits in discussing the similarities and differences between the host country and the students’ home countries. Presbitero and Toledano (2018) and Fakhreldin et al. (2021) reported increases in overall CQ scores following programs that combined courses with experiential activities such as case studies and role-playing. In contrast, Fischer (2011) found a significant improvement only in behavioral CQ, with significant decreases in all other dimensions after role-playing and behavior modification sessions.

## Discussion

This review demonstrates that didactic training effectively enhances cultural competence and cognitive CQ but has limited impact on behavioral and motivational CQ. Experiential training improves overall CQ, though its effects on cognitive and behavioral CQ, as well as cultural competence, remain inconsistent. Mixed-delivery programs provide the most balanced CQ gains; however, inconsistencies persist in motivational and behavioral CQ, with some programs even showing declines in these specific dimensions. In sum, cultural training programs generally lead to positive outcomes, though changes in the cultural competence and/or cultural intelligence of participants are not always statistically significance. Most training programs have been implemented in educational and business settings, with study abroad experiences being particularly common in education. These programs often immerse students in foreign cultures through local events and interactions (Crowne, 2013), with some providing pre-departure courses to set expectations and reduce anxiety (Caligiuri et al., 2001). Previous research supports the positive impact of pre-departure courses on overall CQ (Koo Moon et al., 2012; Chen, 2015), consistent with the findings in this review. Some studies did not find the expected improvements in motivational and behavioral CQ (Alexander et al., 2021; Wood and St. Peters, 2014) following study abroad experiences - a result that has been observed in earlier research (Varela and Gatlin-Watts, 2014). Factors such as high pre-existing motivation, language barriers, or greater opportunities for interactions within the student group may have limited interactions with locals, potentially affecting behavioral CQ (Wood and St. Peters, 2014). Moreover, the cultural distance between home and destination countries could affect the efficacy of training, as larger cultural dissimilarities may lead to adjustment challenges (Kim et al., 2008). For example, Americans often find it challenging to culturally adjust when travelling to Africa (Black et al., 1991), which may impact CQ and competence of Americans following such experiences.

Didactic methods including classroom discussions and instructional materials are often seen as more effective for cognitive CQ (Lopes-Murphy, 2014), and experiential methods for behavioral and motivational CQ, because of the intuitive alignment between methods and dimensions (MacNab, 2012). However, our findings show that experiential methods can also enhance cognitive CQ (e.g., Wood and St. Peters, 2014) while didactic methods can also enhance behavioral CQ (Rehg et al., 2012). Notwithstanding, the review shows that a mixed-delivery approach is the most effective. This aligns with a previous critical review by Littrell and Salas (2005), which emphasizes the benefits of using a variety of delivery methods and activities.

Simulation games, employed in both experiential and mixed delivery programs, produced varied results. Although intended to enhance cultural awareness and CQ, some studies reported no significant improvement in behavioral or cognitive CQ, and some participants may have found these games challenging, overwhelming or counterproductive (Fischer, 2011; see also Bruschke et al., 1993). While they have shown positive effects on cultural competence in some cases (Kratzke and Bertolo, 2013; Hiller and Woźniak, 2009), their impact on CQ remains inconsistent (Fischer, 2011; Bücker and Korzilius, 2015).

Reflective journaling plays an important role in improving self-awareness and intercultural competence by encouraging participants to critically assess their cultural experiences including how cultural values and communication styles influenced their interactions (Alexander et al., 2021; Alexander et al., 2022; Root and Ngampornchai, 2012). However, its effectiveness in improving all dimensions of CQ varies (e.g., Alexander et al., 2021; Azevedo and Shane, 2019) likely due to differences in journaling activities or insufficient guidance (Ryan and Ryan, 2013).

Organizations may consider participants’ specific needs and context to achieve positive training outcomes (Littrell and Salas, 2005). For instance, in global business, focusing on host-country social and business customs through pre-departure, post-arrival, or sequential CCT is effective in preparing expatriate workers (Littrell and Salas, 2006). In education, pre-departure programs help students prepare for studying abroad (Dunlap and Mapp, 2017). In psychiatry, integrating culture- and context-specific knowledge—such as culturally sensitive techniques for interviewing and diagnosing—enhances practitioners’ understanding and effectiveness when working with diverse patients (Harris et al., 2008). Similarly, in sports, tailored programs that address culture and sports relevant knowledge, skills, and communication may help coaches and athletes navigate cultural differences (Borges et al., 2022b). Additionally, effective programs frequently provide online support for expatriates, ensuring instant access to training while keeping costs low (Greengard, 1999, cited in Littrell and Salas, 2005). A cost-benefit analysis could compare cultural training programs with one another, enabling organizations to select the most effective training based on cost and applicability across different settings (Lee and Ervin, 2018). However, to our knowledge, no such analysis currently exists.

### Limitations and future research

In this study we did not include a quality assessment or a risk of bias assessment. This was because its focus was on synthesizing training outcomes rather than evaluating methodological quality. However, this means that the study can provide no suggestions regarding future methodological improvements to the field. Future studies can incorporate needs assessments to tailor cultural training programs to specific organizational and contextual requirements. To enhance evaluation methods, research could combine standardized, validated quantitative measures, such as CQ Scale (Ang et al., 2007) or CQ Sport scale (Borges et al., 2022b), with qualitative feedback, including post-experience reflections, to provide a more comprehensive understanding of cultural competence or CQ outcomes. Future research can implement long-term follow-up assessments (see Harris et al., 2008) with care to include co-variate measures such as migration experience, to better assess the sustained impact of cultural training programs. Finally, with the increasing shift toward digital learning, future studies should continue to explore the effectiveness of online or hybrid cultural training programs.

## Conclusion

This review shows the effectiveness of cross-cultural training programs, particularly those that use diverse activities and mixed-delivery methods, in improving participants’ cultural competence and intelligence. However, further research is needed to develop and evaluate programs tailored specifically for sports settings. It is essential that individuals engaged in multicultural interactions have the tools to effectively manage cultural differences and training programs are generally effective in achieving this.

## Data Availability

The data is publicly available in the Supplementary materials of this manuscript.
