# Supplementary material for: Exploring the impact of cross-cultural training on cultural competence and cultural intelligence: a narrative systematic literature review
Source: Front Psychol. 2025 Apr 7;16:1511788. doi: 10.3389/fpsyg.2025.1511788 (PMC12009937; doi:10.3389/fpsyg.2025.1511788)
Supplement: Supplementary file 1 [file Data_Sheet_1.docx]

**Supplementary Material**

**List of excluded papers**

**Papers not retrieved (n=11)**

1. Boulware-Brown, P.V., 2004. *Cultural competence training with law enforcement personnel: A promising training model*. Regent University.
2. Jauregui, M., 2013. *Cross-cultural training of expatriate faculty teaching in international branch campuses*. Doctoral dissertation. University of Southern California.
3. Moss, F., 2010. *The impact of a cultural immersion experience in building cultural competence of preservice teachers in urban schools*. ProQuest LLC.
4. Pallotta, C.J., 2017. *Cross-cultural competency education at the US Naval Academy*. *US Naval Institute Proceedings*, 143(7), pp.70-73.
5. Lott, T.S., 2012. *Cultural competence: The impact of training on rural child welfare professionals*. Capella University.
6. AbuDagga, A., Weech-Maldonado, R. and Tian, F., 2018. Organizational characteristics associated with the provision of cultural competency training in home and hospice care agencies. *Health Care Management Review*, 43(4), pp.328-337.
7. Acosta-Mosquera, E., Albar Marín, M.J., García-Ramírez, M. and Aguilera-Jiménez, A., 2017. Implementing cultural competence training as a psychopolitical empowerment process. *Journal of Prevention & Intervention in the Community*, 45(1), pp.70-80.
8. Baranova, L., 2018. International educational trends in cross-cultural training. *Порівняльна професійна педагогіка*, 8(2), pp.148-152.
9. King, C.J., Gamble, D., Guiton, G. and Kaul, P., 2023. Student clinical experiences in cross-cultural education. *Southern Medical Journal*, 116(5), pp.390-394.
10. Reitmanova, S., 2011. Cross-cultural undergraduate medical education in North America: theoretical concepts and educational approaches. *Teaching and Learning in Medicine*, 23(2), pp.197-203.
11. Plangsorn, B., Na-Songkhla, J. and Luetkehans, L.M., 2016. Undergraduate students' opinions with regard to ubiquitous MOOC for enhancing cross-cultural competence. *World Journal on Educational Technology: Current Issues*, 8(3), p.210.

**No type of training (n=13)**

1. Lenartowicz, T., Johnson, J.P. and Konopaske, R., 2014. The application of learning theories to improve cross-cultural training programs in MNCs. *The International Journal of Human Resource Management*, 25(12), pp.1697-1719.
2. Kamal Abdien, M. and Jacob, M., 2019. Cross-cultural training and cultural intelligence of hospitality students: a case study in Egypt and Spain. *Journal of Teaching in Travel & Tourism*, 19(3), pp.191-215.
3. Javier, J.R., Hendriksz, T., Chamberlain, L.J. and Stuart, E., 2013. Cross-cultural training in pediatric residency: every encounter is a cross-cultural encounter. *Academic Pediatrics*, 13(6), p.495.
4. Jung, J., 2022. English fluency is not enough: My journey to cultural competency. *Journal of Cancer Education*, 37(5), pp.1574-1576.
5. Kotsaga, E., 2015. Cross-cultural training as a critical factor of cultural intelligence in the hospitality industry. *Tourismos*, 10(2), pp.213-222.
6. Koo Moon, H., Kwon Choi, B. and Shik Jung, J., 2012. Previous international experience, cross‐cultural training, and expatriates' cross‐cultural adjustment: Effects of cultural intelligence and goal orientation. *Human Resource Development Quarterly*, 23(3), pp.285-330.
7. Chen, A.S.Y., 2015. CQ at work and the impact of intercultural training: An empirical test among foreign laborers. *International Journal of Intercultural Relations*, 47, pp.101-112.
8. Bean, R., 2006. Cross-cultural competence and training in Australia. *The Diversity Factor*, 14(1), pp.14-22.
9. Cardon, P.W., 2010. Using films to learn about the nature of cross-cultural stereotypes in intercultural business communication courses. *Business Communication Quarterly*, 73(2), pp.150-165.
10. Cordero, A., 2008. Towards cultural competency with a Latino community: A cross-cultural teaching model. *Journal of Teaching in Social Work*, 28(1-2), pp.165-189.
11. Filmer, T. and Herbig, B., 2020. A training intervention for home care nurses in cross‐cultural communication: An evaluation study of changes in attitudes, knowledge and behaviour. *Journal of Advanced Nursing*, 76(1), pp.147-162.
12. El Achi, D., Brown, A., Huguenard, S., Khan, S., Streff, H. and Nassef, S., 2024. Perceived impact of ethnocultural competency training on genetic counselors' clinical interactions. *Journal of Genetic Counseling*, 33(3), pp.605-614.
13. Gregg, J. and Saha, S., 2006. Losing culture on the way to competence: the use and misuse of culture in medical education. *Academic Medicine*, 81(6), pp.542-547.

**No intervention implementation (n=16)**

1. Farrelly, T. and Lumby, B., 2009. A best practice approach to cultural competence training. *Aboriginal and Islander Health Worker Journal*, 33(5), pp.14-22.
2. Semlali, I., Tamches, E., Singy, P. and Weber, O., 2020. Introducing cross-cultural education in palliative care: focus groups with experts on practical strategies. *BMC Palliative Care*, 19, pp.1-10.
3. Chen, Y.L., 2015. Examining factors in cross-cultural competence development in Taiwan college students. *The Asia-Pacific Education Researcher*, 24, pp.329-340.
4. Riley, L., Howard-Wagner, D. and Mooney, J., 2015. Kinship online: Engaging ‘cultural praxis’ in a teaching and learning framework for cultural competence. *The Australian Journal of Indigenous Education*, 44(1), pp.70-84.
5. Chang, W.W., 2007. Cultural competence of international humanitarian workers. *Adult Education Quarterly*, 57(3), pp.187-204.
6. Friedman, H., Glover, G., Sims, E., Culhane, E., Guest, M. and Van Driel, M., 2013. Cross-cultural competence: Performance-based assessment and training. *Organization Development Journal*, 31(2), p.18.
7. Craig, S., Hull, K., Haggart, A.G. and Perez-Selles, M., 2000. Promoting cultural competence through teacher assistance teams. *Teaching Exceptional Children*, 32(3), p.6.
8. Damron, R.L. and Halleck, G.B., 2007. Generating cross-cultural training data for THE UNIVERSITY GAME. *Simulation & Gaming*, 38(4), pp.556-568.
9. Watson, J.R., 2010. Language and culture training: Separate paths? *Military Review*, March-April, pp.97-105.
10. Dewees, M., 2001. Building cultural competence for work with diverse families: Strategies from the privileged side. *Journal of Ethnic & Cultural Diversity in Social Work*, 9(3-4), pp.33-51.
11. Boucher, N.A. and Johnson, K.S., 2021. Cultivating cultural competence: how are hospice staff being educated to engage racially and ethnically diverse patients? *American Journal of Hospice and Palliative Medicine®*, 38(2), pp.169-174.
12. Thew, D., Smith, S.R., Chang, C. and Starr, M., 2012. The Deaf Strong Hospital program: A model of diversity and inclusion training for first-year medical students. *Academic Medicine*, 87(11), pp.1496-1500.
13. Dabney, K., McClarin, L., Romano, E., Fitzgerald, D., Bayne, L., Oceanic, P., Nettles, A.L. and Holmes Jr, L., 2016. Cultural competence in pediatrics: Health care provider knowledge, awareness, and skills. *International Journal of Environmental Research and Public Health*, 13(1), p.14.
14. Bennett, R., Aston, A. and Colquhoun, T., 2000. Cross‐cultural training: A critical step in ensuring the success of international assignments. *Human Resource Management*, 39(2‐3), pp.239-250.
15. Lee, A., Poch, R., Shaw, M. and Williams, R., 2012. Developing a pedagogy that supports intercultural competence. *ASHE Higher Education Report*, 38(2), pp.45-63.
16. Kripalani, S., Bussey-Jones, J., Katz, M.G. and Genao, I., 2006. A prescription for cultural competence in medical education. *Journal of General Internal Medicine*, 21, pp.1116-1120.

**No outcome measures or irrelevant intervention assessment (n=11)**

1. Ho, M.J., Yao, G., Lee, K.L., Beach, M.C. and Green, A.R., 2008. Cross-cultural medical education: Can patient-centered cultural competency training be effective in non-Western countries? *Medical Teacher*, 30(7), pp.719-721.
2. Mazur, R. and Woodland, R.H., 2017. Evaluation of a cross-cultural training program for Pakistani educators: Lessons learned and implications for program planning. *Evaluation and Program Planning*, 62, pp.25-34.
3. Marovic, Z., 2020. Cross-cultural indigenous training: The South African experience. *Culture & Psychology*, 26(3), pp.605-621.
4. Bai, J., Larimer, S. and Riner, M.E., 2016. Cross-cultural pedagogy: Practical strategies for a successful interprofessional study abroad course. *Journal of the Scholarship of Teaching and Learning*, 16(3), pp.72-81.
5. Staton, L.J., Estrada, C., Panda, M., Ortiz, D. and Roddy, D., 2013. A multimethod approach for cross-cultural training in an internal medicine residency program. *Medical Education Online*, 18(1), p.20352.
6. Lewandowski, C., Andrea, T. and Taylor, C.P., 2021. Student perceptions of cultural competency. *Optometric Education*, 46(2).
7. Haas, M., 2006. Strategies for teaching culture in grades K-8. *Learning Languages*, 11(2), pp.12-17.
8. Iftanti, E., 2018. Assessment model implemented in learning gallery to teach cross-cultural understanding for EFL learners. *Dinamika Ilmu*, 18(1), pp.51-62.
9. Kelly, K., Lee, S.H., Bowen Ray, H. and Kandaurova, M., 2018. Using the photovoice methodology to increase engagement and sharpen students’ analytical skills regarding cultures, lifestyles, and markets internationally. *Marketing Education Review*, 28(2), pp.69-74.
10. Wilcox, K.C., 2009. The impact of student beliefs on the effectiveness of video in developing cross-cultural competence. *Calico Journal*, 27(1), pp.91-100.
11. Mills‐Powell, D. and Worthington, R., 2007. Space for GRRAACCEESS: Some reflections on training for cultural competence. *Journal of Family Therapy*, 29(4), pp.364-367.

**Review articles (n=4)**

1. Downing, R., Kowal, E. and Paradies, Y., 2011. Indigenous cultural training for health workers in Australia. *International Journal for Quality in Health Care*, 23(3), pp.247-257.
2. Sit, A., Mak, A.S. and Neill, J.T., 2017. Does cross-cultural training in tertiary education enhance cross-cultural adjustment? A systematic review. *International Journal of Intercultural Relations*, 57, pp.1-18.
3. Venkataramu, V.N., Vajawat, B., Raghuraman, B.S. and Chaturvedi, S.K., 2021. Cultural competency training for psychiatry residents and mental health professionals: A systematic review. *International Journal of Social Psychiatry*, 67(7), pp.833-839.
4. Klenner, M., Mariño, R., Pineda, P., Espinoza, G. and Zaror, C., 2022. Cultural competence in the nursing, dentistry, and medicine professional curricula: A qualitative review. *BMC Medical Education*, 22(1), p.686.
