# Supplementary material for: Exploring the impact of cross-cultural training on cultural competence and cultural intelligence: a narrative systematic literature review
Source: Front Psychol. 2025 Apr 7;16:1511788. doi: 10.3389/fpsyg.2025.1511788 (PMC12009937; doi:10.3389/fpsyg.2025.1511788)
Supplement: Supplementary file 2 [file Table_1.docx]

**Supplementary Table 1.** Summary of included studies

| **Author (Year)** | **Participants (n)** | **Intervention  (Cross-cultural training)** | **Methodology Experiment design** | **Measurement** | **Main findings** |
| --- | --- | --- | --- | --- | --- |
| 1. Alexander, Ingersoll, Calahan, Miller, Shields, Gipson and Alexander, 2021 | - Students (n=115) | **Training activity:** Cultural learning course and study abroad experience  **Duration:** 9-week course and 3-week study abroad | Quantitative  Mixed design | Cultural Intelligence Scale (CQS) (Ang et al., 2007) | - **Experimental group:** Significant increase in cognitive, metacognitive, behavioral CQ but non-significant increase in motivational CQ. - **Control group:** Non-significant changes across all CQ dimensions, with small increase in metacognitive CQ. |
| 1. Alexander, Ingersoll, Shields, Miller, Gipson, Calahan, DeMaria and Alexander, 2022 | - Students (n=51) | **Training activity:**Cultural development course and study abroad experience   **Duration:**  10-week course (each class 3 hours)  3-weeks and 6-weeks study abroad | Quantitative  Mixed design | Cultural Intelligence Scale (CQS) (Ang et al., 2007) | - **Experimental group:** Significant increase across all CQ dimensions for both groups (3-week and 6-week study abroad). Significant increase in metacognitive CQ of students in the 3-week study abroad program than the students in the 6-week study abroad program. - **Control group:** Non-significant changes across all CQ dimensions. |
| 1. Alexandra, 2018a | - Post-graduate students (n=122) | **Training activity:** 7-stage Experiential CQ Education  **Duration:** 6-8 weeks | Quantitative  Within subject design | Cultural Intelligence Scale (CQS) (Ang et al., 2007) | - Significant increase in overall CQ scores following training. |
| 1. Alexandra, 2018b | - Management students (n=174) | **Training activity:**  7-stage Experiential CQ Education  **Duration:** 6-8 weeks | Quantitative  Within subject design | Cultural Intelligence Scale (CQS) (Ang et al., 2007) | - Significant increase in overall CQ scores following training. |
| 1. Azevedo and Shane, 2019 | - Students and professionals (n=66) | **Training activity:** Courses and activities   **Duration (MBA students):** First weekend: 19 hours  Online sessions: 8 days  Second weekend: 16 hours  **Duration (HR professionals):** 2 days day workshop (16 hours) | Quantitative  Mixed design | Expanded Cultural Intelligence Scale (E-CQS) (Van Dyne, 2012) | - **MBA students:** Significant increase in motivational, metacognitive, and cognitive CQ, with a moderate increase in behavioral CQ. - **HR professionals:** Significant increase in metacognitive and behavioral CQ, with a moderate increase in cognitive CQ and a small increase in motivational CQ. |
| 1. Bücker and Korzilius, 2015 | - Business students (n=81) | **Training activity:** The Ecotonos Game **-** Interactive role play    **Duration:** Pre-defined amount of time for the game | Quantitative  Mixed design | Cultural Intelligence Scale (CQS) (Ang et al., 2007) | - **Within experimental groups:** Significant increase in overall CQ scores and in metacognitive, motivational, and behavioral CQ. Non-significant increase in cognitive CQ. - **Experimental group:** Significant increase in overall CQ scores. - **Control group:** Non-significant increase in overall CQ scores or any CQ dimensions. |
| 1. Dunlap and Mapp, 2017 | - Students (n=25) | **Training activity:** Pre-departure course and experience abroad    **Duration:** Weekly meetings - semester long | Quantitative  Mixed design | Cultural Competence Assessment Instrument (CCAI) (Kelley and Meyers, 1995) | - Students who participated in both the class and study abroad program showed an improvement in their scores from Time 1 to Time 2, with continued improvement by Time 3. - Significant differences in cultural competence scores were observed between students who took the class and those who did not, both at Time 2 and Time 3. - Students who did not take the class or go abroad showed minimal change in scores over the three time points. |
| 1. Eisenberg, Lee, Brück, Brenner, Claes, Mironski and Bell, 2013 | - Study 1:  Students (n=289) - Study 2:  Students (n=185) | **Training activity**:  **Study 1**  Study abroad program with academic-based activities experiential content  **Duration:** 2.5 days course  **Study 2**  Cross-cultural management course (experimental group) and summer school (control group)  **Duration:** 8 weeks (experimental group) and 3 weeks (control group) | Quantitative  Study 1: Within subject design  Study 2:  Mixed design | Cultural Intelligence Scale (CQS) (Ang et al., 2007) | **Study 1:**   - Significant increase in overall CQ scores and metacognitive and cognitive CQ; a non-significant increase in behavioral CQ, and a significant decrease in motivational CQ.   **Study 2:**   - **Experimental group**: Significant increase in overall CQ score and in metacognitive, cognitive, and motivational CQ; a non-significant increase in behavioral CQ. - **Control group:** Non-significant decrease across all CQ dimensions. |
| 1. Engle and Crowne, 2014 | - University students  (n=135) | **Training activity:**Preparation course, tasks, and study abroad experience  **Duration:**  Preparation course (6 hour)  7-12 days abroad experience | Quantitative  Mixed design | Cultural Intelligence Scale (CQS) (Ang et al., 2007) | - **Experimental group:** Significant increase across all CQ dimensions following training. - **Control group:** Non-significant increase across all CQ dimensions following training. |
| 1. Fakhreldin, Youssef and Anis, 2021 | - Students (n=240) | **Training activity:** Cross-cultural management course  **Duration:** Three different semesters | Quantitative & Qualitative  Mixed design | Cultural Intelligence Scale (CQS) (Ang et al., 2007)   Semi-structured interviews with a focus group | - **Experimental group:** Significant increase in CQ scores and its dimensions and significant increase in cultural competence. - **Control group:** Non-significant increase across all CQ dimensions. |
| 1. Fischer, 2011 | - Students (n=107) | **Training activity:**Courses, simulation game (BaFa BaFa), behavior modification session  **Duration:** 4 weeks | Quantitative  Within subject design | Short version of Cultural Intelligence Scale (CQS) (Ang and Van Dyne, 2008)  Self-report | - Significant decrease in cognitive, metacognitive CQ; non-significant decrease in motivational CQ, and non-significant increase in behavioral CQ after training. |
| 1. Harris, McQuery, Raab and Elmore, 2008 | - Psychiatry residents (n=15) | **Training activity:**Educational activities  **Duration:** 9 weeks | Quantitative  Within subject design | The Boston Survey of Culturally Competent Residency Training Practices in Psychiatry Questionnaire (Weiss and Minsky, 1996)  Follow up questionnaire | - **Immediate follow-up:** Significant increase in overall cultural competence scores at the immediate follow-up. - **9-month follow up:** Significant decrease in in ‘Awareness of Privilege’ scores. All the other scores remained statistically unchanged from the immediate follow-up. - Several item scores were lower at the 9-month follow-up compared to scores immediately after the course. |
| 1. Hiller and Woźniak, 2009 | - Students (n=122) | **Training activity:** Lectures, activities, and role play (‘Archivum 2060’) and class simulation games (BaFa BaFa).  **Duration:** A day long | Qualitative  Cross-sectional study | Behavioral Flexibility and Empathy via the role play    Written questionnaire with open-ended questions | Students’ statements concerning the workshop’s outcome:   - Respect for otherness. - Knowledge discovery, behavioral flexibility. - Tolerance for ambiguity. - Empathy. - Behavioral flexibility, communicative awareness respect for otherness. |
| 1. Kirste and Holtbrügge, 2019 | - Students (n=50) | **Training activity:**Online Cultural Intelligence Training, including educational units and exercises  **Duration:** 2 weeks | Quantitative  Mixed design | Cultural Intelligence Scale (CQS) (Ang et al., 2007) | - **Experimental group:** Non-significant changes in overall CQ scores or in any of the dimensions. - **Control group**: Non-significant changes in overall CQ scores or any CQ dimensions. |
| 1. Kratzke and Bertolo, 2013 | - Undergraduate students (n=11) | **Training activity:** Cross-cultural experiential learning exercise, including lectures, classroom simulation exercise (BaFa BaFa).  **Duration:** Lecture overview: 10 minutes Simulation game: 1 hour | Qualitative  Descriptive design | Reflection paper | The three themes were identified related to lessons learned about cultural differences:   - Cultural knowledge and cultural awareness. - Observation and learning. - Cross-cultural communication. |
| 1. Kurpis and Hunter, 2017 | - Students (n=69) | **Training activity:** Experiential CQ Development Activity, including lectures (Primer content) and readings  **Duration:** A semester-long course | Quantitative and Qualitative  Between subject design | Cultural Intelligence Scale (CQS) (Ang et al., 2007)  Post-activity survey  Self-Reported Studies-Based Intercultural Competence    Reflection paper assignment | - **Quantitative results:** International students showed a significant advantage over domestic students in cognitive CQ. The differences along with the other CQ dimensions between the two groups were not significant. - **Qualitative results:** Increased confidence and motivation, increased knowledge about other cultures, broadening of intercultural perspectives, and desire for further experiences. |
| 1. MacNab, 2012 | - Management education participants  (n=373) | **Training activity:** 7-stage Experiential CQ Education   **Duration:** 8 weeks | Quantitative and Qualitative  Within subject design | 1^st^ phase group: Reflection paper  2^nd^ phase group: Cultural Intelligence Scale (Ang et al., 2007) | - **Qualitative results:** The training was both meaningful and relevant in enhancing cultural intelligence (CQ) skills and knowledge. - **Quantitative results:** Significant increase across all CQ dimensions, with cognitive CQ was not involved. |
| 1. Majda, Zalewska-Puchała, Bodys-Cupak, Kurowska and Barzykowski, 2021 | - Nursing students (n=130) | **Training activity: Cultural** Education Training, including practical classes  **Duration:**  Once a week for two weeks (5 hour each session) | Quantitative  Within subject design | Cultural Intelligence Scale (CQS) (Ang et al., 2007)    Cross-Cultural Competence Inventory (CCCI) (Thornson and Ross, 2010) | - **Study 1:** Significant increase across all CQ dimensions but non-significant increase in cultural competence. - **Study 2:** Significant increase across all CQ dimensions; a significant increase in behavioral and motivational CQ. Non-significant increase in cultural competence. |
| 1. Pandey, 2012 | - Students (n=14) | **Training activity:**Workshop  **Duration:** 10 classroom sessions (90 minutes each session) | Qualitative  Exploratory design | Reflection paper | - Students found selected movies very relevant and effective in learning cross-cultural theories, issues and developing cross-cultural competence. - Both instructor’s observations and students’ reactions regarding the effectiveness of movies as classroom learning tool are very positive. |
| 1. Presbitero and Toledano, 2018 | - Global team members (n=252) | **Training activity:**Courses, role playing, case studies    **Duration:** 6 months (one session every month and each session 6 hour) | Quantitative  Within subject design | Cultural Intelligence Scale (CQS) (Ang et al., 2007)    Cross-cultural training survey | - A significant difference between the pre- and post-training scores of CQ. - Significant increase in overall CQ scores after training. |
| 1. Rahayu and Arga, 2019 | - Students (n=99) | **Training activity VBA** (Visual Basic of Application)-based Monopoly Game in Microsoft Excel    **Duration:** Two meetings | Quantitative  Mixed design | Quantitative observation instrument | - **Experimental group:** Significant improvement in students' cross-cultural competency after using a VBA-based Monopoly game in Microsoft Excel as teaching material. - **Control group:** Improvements in students' cross-cultural competency after receiving conventional learning methods. |
| 1. Ramsey and Lorenz, 2016 | - MBA and entrepreneurship students (n=281) | **Training activity:** Cross- cultural management and CQ education    **Duration:** 16 weeks (one-semester) | Quantitative  Mixed design | Cultural Intelligence Scale (CQS) (Ang et al., 2007) | - **Experimental group:** Significant increase in overall CQ scores. - **Control group:** Non-significant changes in overall CQ scores. The experimental group’s CQ scores were significantly higher than those of the control group. |
| 1. Rehg, Gundlach and Grigorian, 2012 | - Military and government civilians (n=110) | **Training activity:** Formal courses    **Duration:** 9 days | Quantitative  Mixed design | Cultural Intelligence Scale (CQS) (Ang et al., 2007) | - **Class 1:** Significant increase in cognitive CQ, with positive but not significant changes in motivational and behavioral CQ. - **Class 2:** Significant increase in both cognitive and behavioral CQ, with a positive but not significant change in motivational CQ. - **Combined results (Class 1 + Class 2):** Significant increase in both behavioral and cognitive CQ, with a positive but not significant increase in motivational CQ. |
| 1. Smith and Bahr, 2014 | - Mental health professionals (n=57) | **Training activity:** Courses and tasks  **Duration:** A full-day course (8-hour) | Quantitative  Mixed design | Multicultural Awareness Knowledge Skills Survey  (MAKKS Survey; D’Andrea et al., 1991 | - Significant increase in cultural competence scores (cultural awareness, knowledge, and skills). |
| 1. Spitzer, 2015 | - Students (n=35) | **Training activity:** Course, activities, tasks    **Duration:** Semester-long course | Qualitative  Within subject design | MyCap (My Cultural Awareness profile) | - Improvement in cross-cultural competence, global awareness, and cultural self-awareness after training. |
| 1. Wood and Peters, 2014 | - MBA students (n=42) | **Training activity:** Cross-cultural study tours and sessions  **Duration:** 11–12-days | Quantitative  Within subject design | Cultural Intelligence Scale (CQS) (Ang et al., 2007) | - Significant increase in cognitive, metacognitive, and motivational CQ after training; non-significant increase in behavioral CQ. |
| 1. Young, Haffejee and Corsun, 2018 | - Students (n=168) | **Training activity:** Diversified mentoring relationships program and tasks  **Duration:** 4 weeks | Quantitative  Mixed design | Cultural Intelligence Scale (CQS) (Ang et al., 2007) | - **Experimental group:** Significant increase in behavioral and metacognitive CQ; non-significant increase in cognitive and motivational CQ. - **Control group:** Significant decrease in cognitive, metacognitive, behavioral, and motivational CQ. - Non-significant difference between the changes in motivational CQ for both the experimental and control group. |

**References**

D’andrea, M., Daniels, J. and Heck, R. (1991) Evaluating the impact of multicultural counseling training, *Journal of Counseling & Development*, 70(1), pp. 143–150. <https://doi.org/10.1002/j.1556-6676.1991.tb01576.x>.

Kelley, C., and Meyers, J. (1995). *Cross-Cultural Adaptability Inventory*. National Computer Systems.

Thornson, C. A., and Ross, K. G. (2010). The Construct & Criterion Validation of the Cross-Cultural Competence Inventory. *Final Report. Defense Equal Opportunity Management Institute (DEOMI)*.

Van Dyne, L., Ang, S., Ng, K. Y., Rockstuhl, T., Tan, M., and Koh, C. (2012). Sub‐Dimensions of the four factor Model of Cultural intelligence: Expanding the conceptualization and measurement of cultural intelligence. *Social and Personality Psychology Compass*, 6(4), pp. 295–313. <https://doi.org/10.1111/j.1751-9004.2012.00429.x>

Weiss, C.I. and Minsky, S., 1996. Self-Assessment Survey Tool. *Trenton, NJ: New Jersey Division of Mental Health Services, Multicultural Advisory Committee*.
