# Supplementary material for: Exploring the impact of cross-cultural training on cultural competence and cultural intelligence: a narrative systematic literature review
Source: Front Psychol. 2025 Apr 7;16:1511788. doi: 10.3389/fpsyg.2025.1511788 (PMC12009937; doi:10.3389/fpsyg.2025.1511788)
Supplement: Supplementary file 3 [file Table_2.docx]

**Supplementary Table 2.** Summary of content and activities used in cross-cultural training programs

| **Author (year)** | **Delivery Method** | **Course Topics, Educational Tasks and Experiential Activities** |
| --- | --- | --- |
| 1. Alexander, Ingersoll, Calahan, Miller, Shields, Gipson and Alexander, 2021 | Mixed delivery | **Course topic(s):**   - Self-awareness, cultural other awareness, managing emotions, and bridging differences, including essential subjects like cultural value dimensions and various communication styles.   **Experiential activities:**   - Role-playing, simulation exercises and debriefing. - Cultural mentors support understanding cultural differences and set expectations for study abroad. - Reflective journaling focuses on intercultural interaction assignments. - 3-week study abroad program (experimental group): Students engage with local cultures and guided reflections. - Summer school program (control group): Students participate in intercultural interactions. |
| 1. Alexander, Ingersoll, Shields, Miller, Gipson, Calahan, DeMaria and Alexander, 2022 | Mixed delivery | **Course topic(s):**   - All four domains of cultural intelligence (CQ) through various assignments   **Educational tasks:**   - Reflective journaling on past cultural interactions and experiences with individuals from diverse cultures.   **Experiential activities:**   - Participants create an action plan based on their reflections and conduct interviews with two people from different cultural backgrounds. - 3-week study abroad program in Europe (experimental group) and 6-week study abroad program in Japan (experimental group): Travel to new locations every 3-4 days. - Summer program (control group): Courses and laboratory work without any cultural training. |
| 1. Alexandra, 2018a | Experiential delivery | **Experiential Activities: 7-stage Experiential CQ Education**   1. Awareness development: A basic knowledge of culture and related processes. 2. Experiential instructions: Learn how to seek contact with members of culturally different groups. 3. Pre-experience check: Submit a description of intended contact to ensure it meets all requirements. 4. New cultural experience: Engage in experiences with a culturally different group. 5. Post-experience and internalization: Write a reflection on successes and challenges related to cultural intelligence (CQ). 6. Feedback and communication: Receive feedback from instructors based on assignment requirements and application of materials. 7. Group discussions and social sharing: Participate in small group discussions to share experiences with peers. |
| 1. Alexandra, 2018b | Experiential delivery | **Experiential Activities: 7-stage Experiential CQ Education**   - 1. Awareness development: A basic knowledge of culture and related processes.   2. Experiential instructions: Learn how to seek contact with members of culturally different groups.   3. Pre-experience check: Submit a description of intended contact to ensure it meets all requirements.   4. New cultural experience: Engage in experiences with a culturally different group.   5. Post-experience and internalization: Write a reflection on successes and challenges related to cultural intelligence (CQ).   6. Feedback and communication: Receive feedback from instructors based on assignment requirements and application of materials.   7. Group discussions and social sharing: Participate in small group discussions to share experiences with peers. |
| 1. Azevedo and Shane, 2019 | Mixed delivery | **Course topic(s):**   - **MBA Students:** Focus on cultural intelligence, mindfulness, authentic leadership, and understanding unconscious biases. - **HR Professionals:** Emphasis on cultural intelligence, self-awareness, internalized moral perspective, balanced processing, and relational transparency.   **Educational tasks – MBA students:**   - Engage in Blackboard discussions and self-study. - Complete an individual foreign film assignment centered on the cultural intelligence (CQ) dimensions. - Maintain a weekly journal to record experiences, insights, and key learnings. - Write an integrative self-reflection essay at the end of the term.   **Experiential activities – MBA Students:**   - A team challenge project including an interview with a manager currently experiencing a cross-cultural challenge in his/her workplace and a team. - Making a presentation to be delivered to the rest of the class.   **Experiential activities – HR Professionals:**   - In-classroom exercises and self-study activities. - The multi-cultural team project: discussion and group exercises. |
| 1. Bücker and Korzilius, 2015 | Experiential delivery | **Experiential activities:** **The Ecotonos Cultural Simulation Game**   - Trainees engage in tasks within a multicultural setting. Participants select cards to create various cultural scenarios and construct a myth explaining the culture's origins, values, and beliefs, emphasizing harmony and survival (p. 98). Initially, participants work on a problem within monocultural groups before introducing their cultures. Participants from diverse backgrounds share their decision-making approaches and cultural norms, diagramming their communication patterns to present to other groups. |
| 1. Dunlap and Mapp, 2017 | Mixed delivery | **Educational tasks:**   - Seminar and group discussions. - Prepare in-class presentations about one’s own culture and a short reflection paper. - Follow a news source from the host country and present an overview of its cultural context. - Utilize the book *Charting a Hero's Journey* for in-depth critical reflections.     **Experiential activities:**   - International and domestic internships. - Reflective assignments abroad and sharing experiences with peers to enhance learning. |
| 1. Eisenberg, Lee, Brück, Brenner, Claes, Mironski and Bell, 2013 | Mixed delivery | **Course topic(s):**   - Cultural dimensions and definitions of culture, divided into two parts: common general cultural elements and region-specific cultural elements corresponding to where the students in that group are going to study.   **Experiential activities:**   - Study 1: Simulation games, interaction with nationals from the target culture, and cultural self-awareness exercises. - Study 2: Summer school international marketing program and a competitive project in culturally diverse teams consisting of 4–5 members. Cross-cultural management issues were not included in the curriculum. |
| 1. Engle and Crowne, 2014 | Mixed delivery | **Educational tasks: Pre-trip preparation**   - Readings about the target country’s history and culture and other relevant background information. - Worksheets about films. - Film tasks focus on the history and culture of the target country. - A debriefing meeting upon returning to the country and discussions of their overall experiences. - Writing 'mini cultural cases' for the students who would be attending the similar short-term international experiences.   **Experiential activities:**   - Short term abroad program for 7-12 days, spending time in a foreign country. |
| 1. Fakhreldin, Youssef and Anis, 2021 | Mixed delivery | **Course topic(s):**   - International business course. - Management of multinational companies' course. - The principal aspects of the management styles course.     **Educational activities:**   - Case studies, blended learning techniques, and videos clips inside and outside the classroom.   **Experiential activities:**   - On-hand activities, analysis of case studies, role playing, discussions, and developing videos. - Active learning activities, including a mix of cognitive and constructionist approaches to learning. |
| 1. Fischer, 2011 | Mixed delivery | **Course topic(s):**   - The course includes cultural content relevant to organizational psychology, including four lectures focused on culture theory, dimensions of culture, and their relevance for organizational psychologists and cultural competence. |
|  |  | **Experiential activities:**   - BaFa simulation game. - Behavior modification session that demonstrates the Excel training program designed to teach basic behavioral competencies for approaching a lecturer in Malaysia. |
| 1. Harris, McQuery, Raab and Elmore, 2008 | Didactic delivery | **Course topic(s):**   - (1) Defining culture and cultural competence; (2) the cultural genogram; (3) cultural identity development; (4) privilege; (5) explanator models of illness; (6) acculturation and the immigration experience; (7) cultural transference and countertransference; (8) modelling discussion and resolution of cross-cultural misunderstandings; and (9) culturally sensitive interviewing, diagnosis and review.   **Educational tasks:**   - Brief large group lectures, discussion of case vignettes, small group discussion, and in-session demonstration of clinical skills. |
| 1. Hiller and Woźniak, 2009 | Mixed delivery | **Course topic(s):**   - Basic theoretical introduction focusing on culture-general knowledge and culture-specific knowledge.   **Educational tasks:**   - Case studies.   **Experiential activities:**   - Cultural simulations, role-playing, critical incidents and world café, mediation exercises as well as exercises from tolerance and diversity training. - ‘Archivum 2060’ role-play and classical simulation games such as ‘BaFa BaFa’. |
| 1. Kirste and Holtbrügge, 2019 | Didactic delivery | **Educational tasks:**   - Four educational online units, each containing two or three exercises: (1) We should focus on What Matters; (2) Being on Time; (3) Whose Job is it Really? and (4) Interpretation. - Interactive videos, surveys, tests, and Shareable Content Object Reference Model (SCORM) modules using a learning authoring toolkit for PowerPoint. |
| 1. Kratzke and Bertolo, 2013 | Mixed delivery | **Course topic(s):**   - Cultural awareness and cultural competence. - Cross-cultural BaFa BaFa classroom simulation exercise overview.   **Educational tasks:**   - Reflective writing assignments.   **Experiential activities:**   - Cross-cultural BaFa BaFa classroom simulation exercise: Students were randomly assigned the next week in the classroom to Alpha and Beta cultures. The new group received a 10- minute orientation to learn the new culture, values, and expected norms - use of informal lecture, CD audio, and notes on an easel or classroom whiteboard for easy reference. - Students were given five minutes to practice the new rules, and each facilitator helped students to remember the new rules during the practice. Students interacted with Alpha or Beta culture and participated as visitors. The groups exchanged visitors, and each visitor shared his/her observations with the group. |
| 1. Kurpis and Hunter, 2017 | Mixed delivery | **Course topic(s):**   - The ‘Primer’ theoretical component focuses on dimensions of culture, types of cultural environments, self-reference criterion and ethnocentrism.   **Educational tasks:**   - Prime content: Readings and discussions, and case studies from ‘The Wall Street Journal’ and similar periodicals. - International students: In-class discussions of cultural stereotypes and biases and of ways of dealing with culture-based stereotyping.   **Experiential activities:**   - Cross-cultural consumer behavior interview with international students about the differences and similarities in consumer behavior between the United States and the home countries of these international students. - Writing a reflection paper based on the interviews and sharing their experiences during in-class discussions. |
| - 1. MacNab, 2012 | Experiential delivery | **Experiential activities: 7-stage Experiential CQ Education**   - 1. Awareness development: A basic knowledge of culture and related processes.   2. Experiential instructions: Learn how to seek contact with members of culturally different groups.   3. Pre-experience check: Submit a description of intended contact to ensure it meets all requirements.   4. New cultural experience: Engage in experiences with a culturally different group.   5. Post-experience and internalization: Write a reflection on successes and challenges related to cultural intelligence (CQ).   6. Feedback and communication: Receive feedback from instructors based on assignment requirements and application of materials.   7. Group discussions and social sharing: Participate in small group discussions to share experiences with peers. |
| - 1. Majda, Zalewska-Puchała, Bodys-Cupak, Kurowska and Barzykowski, 2021 | Experiential delivery | **Experiential activities:**   - Intercultural communication workshops covering culture related topics. - Simulation, role-playing, visualization, cases, didactic games, brainstorming. |
| - 1. Pandey, 2012 | Didactic delivery | **Course topic(s):**   - Managing cross-cultural issues. - A seminar-cum-workshop, including lectures and video cases related to the given movies (Outsourced and My Big Fat Greek Wedding).   **Educational tasks:**   - Readings selected chapters from reference books, case studies, and recommended research papers before each session. |
| - 1. Presbitero and Toledano, 2018 | Mixed delivery | **Course topic(s):**   - How different cultures operate and function.   **Educational tasks:**   - Case studies analyzing various situations and identifying the best approach to successfully deal with cross-cultural situations.   **Experiential activities:**   - Role-plays simulating how to effectively interact with clients from different cultural backgrounds. |
| - 1. Rahayu and Arga, 2019 | Experiential delivery | **Experiential Activities: VBA-based Monopoly Game in Microsoft Excel**   - The game includes questions that students must answer when they step on the monopoly box, aiming to teach honesty, sportsmanship, mutual respect, communication, and cooperation. - The control group’s conventional learning includes worksheets in the form of puzzles. |
| - 1. Ramsey and Lorenz, 2016 | Didactic delivery | **Course topic(s):**   - The terminology of cross-cultural management course and what the literature on the topic had found.   **Educational task:**   - International business current events, analyzing 4-6 video clips from the sources of Financial Times and Wall Street Journal. |
| - 1. Rehg, Gundlach and Grigorian, 2012 | Didactic delivery | **Course topic(s):**   - Cross-cultural awareness course focuses on comparing US values with other world views, and how culture affects behavior, perspective, and the ability to function in a dissimilar culture. - Specific aspects of contingency contracting procedures, and laws and regulations. |
| - 1. Smith and Bahr, 2014 | Didactic delivery | **Course topic(s):**   - (1) Awareness, (2) Overview of cultural competence, and (3) Hispanic knowledge and skills. - A presentation on cultural characteristics of the historic population; acquisition of a second language; patterns of Hispanic parental involvement in the school; best practices in working with translators; and verbal and nonverbal communication with Hispanic students and families.   **Educational tasks:**   - Self-reflective and interactive exercises. - Small group discussions, debriefing within a large group, sharing thoughts about one’s self-identity or listening to observations of colleagues. |
| - 1. Spitzer, 2015 | Didactic delivery | **Course topic(s):**   - Demographic trends in the US (and in students’ own towns or cities), the acquisition of language and culture, educational, linguistic, and cultural accommodations. - Other cultural traditions (their own and others from presentations and projects), culture shock and the process of acculturation (recorded experiences of respondents to student-created surveys), building relationships across cultures and cross-cultural communication (reflective papers), moving from fear toward tolerance and understanding (documented in final paper).   **Educational tasks:**   - Journal assignments based on readings which explore cultural characteristics and values. - Reactions to presentations made to class by professors in the college from other cultural backgrounds. - A cultural self-identification oral presentation. - A final project and the creation of survey and administration of interviews with people who are part of a different ethnic group and born outside this country. - A final reflective essay explaining what had been learned in the course. |
| - 1. Wood and Peters, 2014 | Experiential delivery | **Experiential activities:**   - Cross-cultural study tours: (1) Hong Kong and Mainland China, (2) Italy and Germany and (3) Costa Rica. - This program includes visiting 8–12 businesses and visiting several culturally significant sites, eating in local restaurants, interacting with local nationals and other activities to give them exposure to the local culture. - Question and answer sessions. - A study tour reflective journal. |
| - 1. Young, Haffejee and Corsun, 2018 | Mixed delivery | **Educational tasks:**   - Write clear descriptions of the roles needed to successfully execute the multicourse food and wine pairing dinner for their protégés. - Conduct skills assessments of their protégés. - Develop interview protocols and conduct interviews with refugees. - Complete the selection process and design training to prepare protégés for dinner. - Perform performance appraisals by observing their protégés’ performance during the dinner.   **Experiential activities:**   - Before starting the assigned work, spend 2-3 hours with your protégés. - Collaborate with refugees to complete required course assignments that involve experiential learning, allowing students to actively engage in activities with their protégés. |
