# Supplementary material for: Exploring the impact of cross-cultural training on cultural competence and cultural intelligence: a narrative systematic literature review
Source: Front Psychol. 2025 Apr 7;16:1511788. doi: 10.3389/fpsyg.2025.1511788 (PMC12009937; doi:10.3389/fpsyg.2025.1511788)
Supplement: Supplementary file 4 [file Table_3.docx]

**Supplementary Table 3.** Summary of qualitative results

| **Author (Year)** | **Method of training** | **Measurement** | **Sample size (n)** | **Key findings** |
| --- | --- | --- | --- | --- |
| **Fakhreldin, Youssef and Anis, 2021** | Mixed delivery | Semi-structured interviews with a focus group | 240 | The program was effective in improving cultural competence and conflict management abilities with following outcomes:   - An improvement in skills and attributes associated with metacognitive, motivational, and behavioral cultural intelligence (CQ). - A higher level of cultural intelligence (CQ) levels of students following the training. |
| **Hiller and Woźniak, 2009** | Mixed delivery | Feedback - written questionnaire with open-ended questions | 122 | The program was effective in enhancing intercultural competence. The following classifications are based on students’ statements regarding the workshop's outcomes:   - The importance of tolerance (Knowledge discovery, behavioral flexibility). - Readiness to take distance from the own point of view (Respect for otherness). - There is no need to be afraid of contact with the unknown/strangers (Tolerance for ambiguity). - Empathy and change of perspective (Empathy). - Learning to make compromises (Behavioral flexibility, communicative awareness respect for otherness). |
| **Kratzke and Bertolo, 2013** | Mixed delivery | Reflective writing assignment | 11 | The program was effective in enhancing cultural competence. Three themes were identified related to lessons learned about cultural differences:   - Cultural knowledge and cultural awareness. - Observation and learning. - Cross-cultural communication. |
| **Kurpis and Hunter, 2017** | Mixed delivery | Reflection paper assignment | 69 | The program was effective in enhancing cultural intelligence. Four themes emerged from the thematic analysis of the students’ reflection papers:   - Increase in confidence and motivation. - Increase in specific knowledge of another culture. - Broadening of intercultural perspectives, emergence of ethnorelative attitudes. - Desire for further experiences of this nature. |
| **MacNab, 2012** | Experiential delivery | Reflective evaluations | 373 | The program was effective in enhancing cultural intelligence. Below is a summary of participant responses to evaluation questions:   1. Question: “I would recommend this assignment and experience for others interested in improving their cross-cultural skills.” (*M* = 4.127, *SD* = 0.758) 2. Question: “This assignment and experience are meaningful.” (*M* = 4.197, *SD* = 0.752) 3. Question: “This assignment and experience allowed me to better understand abstract concepts related to cultural interaction.” (*M* = 4.191, *SD* = 0.761) 4. Question: “I consider this assignment an intellectual growth experience.” (*M*= 4.149, *SD*= 0.779) 5. Question: “This assignment and experience should be part of a cross-cultural management course.” (*M* = 4.339, *SD* = 0.765) |
| **Pandey, 2012** | Didactic delivery | Reflection notes | 14 | The classroom learning tool was effective in improving cultural competence by providing knowledge on the following concepts:   - Culture shock. - Acculturation process. - Cultural adaptation. - Culture and intercultural communication. - Cultural adjustment. - Family culture. - Influence of family culture on self and personality. - Cross-cultural competence, cultural intelligence. - Differences between Eastern and Western cultures. |
| **Smith and Bahr, 2014** | Didactic delivery | Open ended questions of the effectiveness of training | 57 | The program was effective in improving cultural competence with following outcomes:   - Increase in cultural awareness of one’s own cultural background, beliefs, attitudes, and biases. - Increase in cultural knowledge of cultural group differences. - Increase in awareness about own biases, prejudices, and assumptions. - Increase in cultural communication. |
| **Spitzer, 2015** | Didactic delivery | MyCap (My Cultural Awareness pile) | 35 | The program was effective in improving cultural competence with following outcomes:   - Increase in knowledge about cross-cultural competence. - Increase in global awareness. - Increase in cultural self-awareness. |
