# Supplementary material for: Exploring the impact of cross-cultural training on cultural competence and cultural intelligence: a narrative systematic literature review
Source: Front Psychol. 2025 Apr 7;16:1511788. doi: 10.3389/fpsyg.2025.1511788 (PMC12009937; doi:10.3389/fpsyg.2025.1511788)
Supplement: Supplementary file 5 [file Table_4.docx]

**Supplementary Table 4.** Summary of quantitative results

| **Author (Year)** | **Training method** | **Measurement** | **Sample size (n)** | **Statistical analysis methods and**  **key outcomes** |
| --- | --- | --- | --- | --- |
| 1. **Alexander, Ingersoll, Calahan, Miller, Shields, Gipson and Alexander, 2021** | Mixed delivery | Cultural Intelligence Scale (CQS) | - Experimental group (n=53) - Control group (n=62) | - - - - **Statistical analysis method:** Multi-level model of change.       - **Experimental group**: Significant increase across all CQ dimensions from Time 1 to Time 2.  \| **CQ Dimension** \| **Time 1**  **(M ± SD)** \| **Time 2**  **(M ± SD)** \| **Effect Size (d)** \| **Confidence Interval (95% CI)** \| \| --- \| --- \| --- \| --- \| --- \| \| **Experimental Group** \|  \|  \|  \|  \| \| Motivational CQ \| 5.86 **±** 0.66 \| 5.98 **±** 0.56 \| 0.20 \| [CI -0.344-0.736] \| \| Cognitive CQ \| 3.90 **±** 1.23 \| 4.80 **±** 0.85 \| 0.85 \| [CI 0.289-1.414] \| \| Metacognitive CQ \| 5.36 **±** 0.77 \| 6.16 **±** 0.58 \| 1.17 \| [CI 0.591- 1.757] \| \| Behavioral CQ \| 4.77 **±** 1.03 \| 5.75 **±** 0.76 \| 1.08 \| [CI 0.506 - 1.659] \|  - **Control group**: Non-significant changes across all CQ dimensions, with a decrease in motivational CQ and small increase in cognitive, metacognitive, and behavioral CQ.  \| **CQ Dimension** \| **Time 1**  **(M ± SD)** \| **Time 2**  **(M ± SD)** \| **Effect Size (d)** \| **Confidence**  **Interval (95% CI)** \| \| --- \| --- \| --- \| --- \| --- \| \| **Control Group** \|  \|  \|  \|  \| \| Motivational CQ \| 5.64 **±** 0.78 \| 5.56 **±** 0.90 \| 0.09 \| [CI –0.593 - 0.403] \| \| Cognitive CQ \| 4.40 **±** 1.09 \| 4.53 **±** 1.19 \| 0.11 \| [CI –0.384 - 0.612] \| \| Metacognitive CQ \| 5.09 **±** 0.83 \| 5.39 **±** 0.88 \| 0.35 \| [CI –0.151-0.852] \| \| Behavioral CQ \| 4.45 **±** 1.05 \| 4.57 **±** 1.17 \| 0.11 \| [CI –0.39 -0.606] \| |
| 1. **Alexander, Ingersoll, Shields, Miller, Gipson, Calahan, DeMaria and Alexander, 2022** | Mixed delivery | Cultural Intelligence Scale (CQS) | - 3-week study abroad (n=26) - 6-week study abroad (n=25) - Control group (n= NA) | - - - - **Statistical analysis method:** Multi-level model of change.   **3-week Study Abroad Program**   \| **CQ Dimension** \| **Time 1**  **(M ± SD)** \| **Time 2**  **(M ± SD)** \| **Increase** \| **Effect Size (d)** \| **Confidence Interval (CI 95%)** \| \| --- \| --- \| --- \| --- \| --- \| --- \| \| Motivational CQ \| 5.53 **±** 0.24 \| 6.00 **±** 0.69 \| +0.47 \| 0.91 \| [CI 0.102-1.717] \| \| Cognitive CQ \| 3.46 **±** 0.49 \| 5.10 **±** 0.36 \| +1.64 \| 3.81 \| [CI 2.524 - 5.105] \| \| Metacognitive CQ \| 4.84 **±** 0.40 \| 5.86 **±** 0.26 \| +1.02 \| 3.02 \| [CI 1.898 - 4.149] \| \| Behavioral CQ \| 4.09 **±** 0.49 \| 5.55 **±** 0.36 \| +1.46 \| 3.39 \| [CI 2.195- 4.597] \|  - - - - **3-week study abroad group**: Significant increase across all CQ dimensions from Time 1 (t1) to Time 2 (t2).   **6-week Study Abroad Program**   \| **CQ Dimension** \| **Time 1  (M ± SD)** \| **Time 2  (M ± SD)** \| **Increase** \| **Effect Size (d)** \| **Confidence Interval (CI 95%)** \| \| --- \| --- \| --- \| --- \| --- \| --- \| \| Motivational CQ \| 5.77 **±** 0.18 \| 6.16 **±** 0.26 \| +0.39 \| 1.74 \| [CI 0.823 -2.665] \| \| Cognitive CQ \| 4.02 **±** 0.34 \| 5.26 **±** 0.36 \| +1.24 \| 3.54 \| [CI 0.823 -2.665] \| \| Metacognitive CQ \| 5.47 **±** 0.29 \| 5.97 **±** 0.32 \| +0.50 \| 1.64 \| [CI 2.285 -4.798] \| \| Behavioral CQ \| 4.61 **±** 0.54 \| 6.06 **±** 0.38 \| +1.45 \| 3.11 \| [CI 1.941- 4.27] \|  - **6-week study abroad group**: Significant increase across all CQ dimensions from Time 1 (t1) to Time 2 (t2).   **Control Group**   \| **CQ Dimension** \| **Time 1**  **(M ± SD)** \| **Time 2**  **(M ± SD)** \| **Increase** \| **Effect Size (d)** \| \| --- \| --- \| --- \| --- \| --- \| \| Motivational CQ \| 5.35 **±** 0.99 \| 5.36 **±** 0.99 \| +0.01 \| 0.01 \| \| Cognitive CQ \| 4.45 **±** 1.29 \| 4.46 **±** 1.29 \| +0.01 \| 0.01 \| \| Metacognitive CQ \| 5.07 **±** 1.27 \| 5.07 **±** 1.27 \| 0 \| 0.00 \| \| Behavioral CQ \| 4.60 **±** 1.58 \| 4.58 **±** 1.59 \| -0.02 \| 0.01 \|  - **Control group**: Non-significant changes across all CQ dimensions from Time 1 (t1) to Time 2 (t2). Very small increase in motivational, cognitive, and behavioral CQ, with a non-significant increase in metacognitive CQ. - **Note:** No data available on the control group sample size. |
| 1. **Alexandra, 2018a** | Experiential delivery | Cultural Intelligence Scale (CQS) | n=122 | - **Statistical analysis method:** Paired sample t-test. Significant increase in overall CQ scores.  \| **Metric** \| **Pre**  **(M ± SD)** \| **Post**  **(M ± SD)** \| **Increase** \| **Significance** \| **Effect size (d)** \| **Confidence Interval (CI 95%)** \| \| --- \| --- \| --- \| --- \| --- \| --- \| --- \| \| Overall CQ \| 3.69 ± 0.43 \| 4.06 ± 0.40 \| 0.37 \| *p* < 0.0001 \| 0.89 \| [CI 0.519- 1.263] \| |
| 1. **Alexandra, 2018b** | Experiential delivery | Cultural Intelligence Scale (CQS) | n=174 | - **Statistical analysis method:** Paired sample t-test. Significant increase in overall CQ scores.  \| **Metric** \| **Pre**  **(M ± SD)** \| **Post**  **(M ± SD)** \| **Increase** \| **Significance** \| **Effect size (d)** \| **Confidence Interval (CI 95%)** \| \| --- \| --- \| --- \| --- \| --- \| --- \| --- \| \| Overall CQ \| 3.69 ± 0.44 \| 4.01 ± 0.40 \| 0.32 \| *p* < 0.0001 \| 0.76 \| [CI 0.453-1.069] \| |
| 1. **Azevedo and Shane**, **2019** | Mixed delivery | Expanded Cultural Intelligence Scale (E-CQS) | MBA students  (n=40)  HR professionals  (Motivational CQ: n=70  Metacognitive CQ: n=64  Cognitive CQ: n=72  Behavioral CQ: n=68) | - **Statistical analysis method:** Wilcoxon signed rank-test. - Significant increase across all CQ dimensions for both students and professionals. - **MBA students:** Significant increase in motivational, metacognitive, and cognitive CQ, with a moderate increase in behavioral CQ.   **MBA Students**   \| **CQ Dimension** \| **Pre  Mdn** \| **Post  Mdn** \| **Z-value** \| **p-value** \| **Effect Size (r)** \| **Improvement** \| \| --- \| --- \| --- \| --- \| --- \| --- \| --- \| \| Motivational CQ \| 5.67 \| 6.22 \| -3.402 \| 0.001 \| 0.54 \| Large \| \| Metacognitive CQ \| 5.67 \| 6.56 \| -3.198 \| 0.001 \| 0.51 \| Large \| \| Cognitive CQ \| 4.60 \| 5.85 \| -3.922 \| 0.000 \| 0.62 \| Large \| \| Behavioral CQ \| 5.56 \| 6.39 \| -2.802 \| 0.005 \| 0.44 \| Moderate \|   **HR Professionals**   \| **CQ Dimension** \| **Pre Mdn** \| **Post** **Mdn** \| **Z-value** \| **p-value** \| **Effect**  **Size (r)** \| **Improvement** \| \| --- \| --- \| --- \| --- \| --- \| --- \| --- \| \| Motivational CQ \| 5.28 \| 5.61 \| -2.355 \| 0.019 \| 0.28 \| Small \| \| Metacognitive CQ \| 4.89 \| 5.22 \| -4.091 \| 0.000 \| 0.51 \| Large \| \| Cognitive CQ \| 3.20 \| 3.80 \| -3.191 \| 0.001 \| 0.38 \| Moderate \| \| Behavioral CQ \| 4.28 \| 5.28 \| -4.311 \| 0.000 \| 0.52 \| Large \|  - **HR professionals:** Significant increase in metacognitive and behavioral CQ, with a moderate increase in cognitive CQ and a small increase in motivational CQ. - **Note:** Confidence intervals are not provided because the Wilcoxon signed-rank test is non-parametric, relying on ranks rather than means and standard deviations, and does not assume normality. |
| **6. Bücker and Korzilius, 2015** | Experiential delivery | Cultural Intelligence Scale (CQS) | Experimental group (n=66)  Control group (n=15) | **Between Experimental and Control Group**   \| **CQ Dimension** \| **Group** \| **Time 1  (M ± SD)** \| **Time 2  (M ± SD)** \| **Effect size (d)** \| **Confidence Interval**  **(CI %95)** \| \| --- \| --- \| --- \| --- \| --- \| --- \| \| Overall CQ \| Experimental \| 5.07 **±** 0.68 \| 5.30 **±** 0.62 \| 0.35 \| [CI – 0.133 – 0.849 \| \|  \| Control \| 4.92 **±** 0.65 \| 4.89 **±** 0.81 \| -0.04 \| [CI -1.053 - 0.971] \| \| Metacognitive CQ \| Experimental \| 5.30 **±** 0.83 \| 5.64 **±** 0.68 \| 0.45 \| [CI 0.04 – 0.937] \| \|  \| Control \| 5.33 **±** 0.74 \| 5.28 **±** 0.83 \| -0.06 \| [CI -1.076 – 0.949] \| \| Cognitive CQ \| Experimental \| 4.46 **±** 0.89 \| 4.63 **±** 0.86 \| 0.19 \| [CI -0.289 – 0.6878] \| \|  \| Control \| 4.18 **±** 0.75 \| 4.10 **±** 0.84 \| -0.10 \| [CI -1.113 – 0.912] \| \| Motivational CQ \| Experimental \| 5.74 **±** 0.61 \| 5.89 **±** 0.62 \| 0.24 \| [CI -0.24 – 0.728] \| \|  \| Control \| 5.44 **±** 0.83 \| 5.46 **±** 0.66 \| 0.027 \| [CI -0.986 - 1.039] \| \| Behavioral CQ \| Experimental \| 4.94 **±** 0.94 \| 5.24 **±** 0.87 \| 0.33 \| [CI -0.155-0.871] \| \|  \| Control \| 4.96 **±** 1.32 \| 4.95 **±** 1.37 \| -0.01 \| [CI -1.02 – 1.005] \|  - **Statistical analysis method:** Independent sample t-test. - **Experimental group:** Significant increase in overall CQ scores and in metacognitive, motivational, and behavioral CQ, with non-significant changes in cognitive CQ. The experimental group had a significant increase in overall CQ scores than the control group. - **Control group:** Non-significant changes in overall CQ or any of the CQ dimensions.   **Within Experimental Group**   \| **CQ Dimension** \| **Time 1  (M ± SD)** \| **Time 2  (M ± SD)** \| **Increase  (M ± SD)** \| **Significance** \| **Effect size (d)** \| **Confidence Interval (CI %95)** \| \| --- \| --- \| --- \| --- \| --- \| --- \| --- \| \| Overall CQ \| 5.07 **±** 0.67 \| 5.30 **±** 0.62 \| 0.23 **±** 0.47 \| Significant increase \| 0.35 \| [CI -0.133 - 0.843] \| \| Metacognitive CQ \| 5.30 **±** 0.83 \| 5.64 **±** 0.68 \| 0.34 **±** 0.74 \| Significant increase \| 0.45 \| [CI -0.04 - 0.937] \| \| Cognitive CQ \| 4.46 **±** 0.89 \| 4.63 **±** 0.86 \| 0.17 **±** 0.78 \| Non-significant change \| 0.19 \| [CI -0.289 - 0.678] \| \| Motivational CQ \| 5.74 **±** 0.61 \| 5.89 **±** 0.62 \| 0.15 **±** 0.39 \| Significant increase \| 0.24 \| [CI-0.24 - 0.728] \| \| Behavioral CQ \| 4.94 **±** 0.94 \| 5.24 **±** 0.87 \| 0.30 **±** 0.81 \| Significant increase \| 0.33 \| [CI-0.155 - 0.817] \|  - **Statistical analysis method:** Paired sample t-test. - Significant increase in overall CQ scores and in metacognitive, motivational, and behavioral CQ; with a non-significant increase in cognitive CQ. |
| 1. **Dunlap and Mapp, 2017** | Mixed delivery | Cultural Competence Assessment Instrument  (CCAI) | Time 1 (n=23)  Time 2 (n=20)  Time 3 (n=21) | - **Statistical analysis method**: Independent sample t-test. - Non-participating students showed minimal change, with a slight decrease in scores from Time 1 to Time 2 and a moderate increase from Time 2 to Time 3. - All participating students showed an overall positive change, with scores increasing from Time 1 to Time 2 and an additional increase from Time 2 to Time 3. - Students who the class and went abroad showed the largest positive change from Time 1 to Time 2 and continuing to increase from Time 2 to Time 3. - Students who took the class but did not go abroad showed a decrease in scores from Time 1 to Time 2, followed by a positive change from Time 2 to Time 3.  \| **Group** \| **t1 Score** \| **t2 Score** \| **t3 Score** \| **Change from  t1 to t2** \| **Change from  t2 to t3** \| \| --- \| --- \| --- \| --- \| --- \| --- \| \| **Non-participating** \| 224.88 \| 224.31 \| 229.42 \| -0.57 (Decrease) \| +5.11 (Increase) \| \| **All participating** \| 237.11 \| 239.03 \| 244.42 \| +1.92 (Increase) \| +5.39 (Increase) \| \| **Participating and abroad** \| 226.89 \| 233.05 \| 240.71 \| +6.16 (Increase) \| +7.66 (Increase) \| \| **Participating and not abroad** \| 255.00 \| 249.50 \| 250.90 \| -5.50 (Decrease) \| +1.40 (Increase) \|   **Note:** No data available for mean scores and standard deviation scores. |
| 1. **Eisenberg, Lee, Brück, Brenner, Claes, Mironski and Bell, 2013** | Mixed delivery | Cultural Intelligence Scale (CQS) | Study 1:  (n=289)    Study 2:  Experimental group (n=150)  Control group (n=35) | **Study 1 Results**   - **Statistical analysis method:** Paired sample t-test. - Significant increase in overall CQ scores and metacognitive and cognitive CQ; a non-significant increase in behavioral CQ, and a significant decrease in motivational CQ.  \| **CQ Dimension** \| **Pre-M** \| **Post-M** \| **Increase (M)** \| **p-value** \| **Effect Size (d)** \| \| --- \| --- \| --- \| --- \| --- \| --- \| \| Overall CQ \| 4.83 \| 5.01 \| +0.18 \| < .001 \| 0.28 \| \| Metacognitive CQ \| 4.71 \| 5.12 \| +0.39 \| < .001 \| 0.43 \| \| Cognitive CQ \| 4.18 \| 4.55 \| +0.37 \| < .001 \| 0.43 \| \| Motivational CQ \| 5.74 \| 5.56 \| -0.18 \| < .001 \| -0.21 \| \| Behavioral CQ \| 4.83 \| 4.93 \| +0.10 \| ns \| 0.11 \|   **Note:** No data available for standard deviation scores.  **Study 2 Results**   - **Statistical analysis method**: Paired sample t-test. - **Experimental group:** Significant increase in overall CQ scores and in metacognitive, cognitive, and motivational CQ; non-significant increase in behavioral CQ. - **Control group:** Non-significant decrease across all CQ dimensions.  \| **CQ Dimension** \| **Group** \| **Pre-M** \| **Post-M** \| **Increase (M)** \| **p-value** \| **Effect Size (d)** \| \| --- \| --- \| --- \| --- \| --- \| --- \| --- \| \| Overall CQ \| Experimental \| 4.96 \| 5.20 \| +0.24 \| < .001 \| 0.35 \| \| Metacognitive CQ \| Experimental \| 5.20 \| 5.54 \| +0.34 \| < .001 \| 0.44 \| \| Cognitive CQ \| Experimental \| 4.20 \| 4.46 \| +0.26 \| < .01 \| 0.26 \| \| Motivational CQ \| Experimental \| 5.58 \| 5.77 \| +0.19 \| < .01 \| 0.25 \| \| Behavioral CQ \| Experimental \| 4.87 \| 5.01 \| +0.14 \| ns \| 0.14 \|  \| **CQ Dimension** \| **Group** \| **Pre-M** \| **Post-M** \| **Increase (M)** \| **p-value** \| **Effect Size (d)** \| \| --- \| --- \| --- \| --- \| --- \| --- \| --- \| \| Overall CQ \| Control \| 5.30 \| 5.16 \| -0.14 \| ns \| - \| \| Metacognitive CQ \| Control \| 5.56 \| 5.35 \| -0.21 \| ns \| - \| \| Cognitive CQ \| Control \| 4.95 \| 4.86 \| -0.09 \| ns \| - \| \| Motivational CQ \| Control \| 5.73 \| 5.53 \| -0.20 \| ns \| - \| \| Behavioral CQ \| Control \| 5.07 \| 5.13 \| -0.17 \| ns \| - \|   **Note:** No data available for standard deviation scores. |
| 1. **Engle and Crowne, 2014** | Mixed delivery | Cultural Intelligence Scale (CQS) | Experimental group  (n=105)  Control group (n=30) | - **Statistical analysis method**: Paired sample t-test. - **Experimental group:** Significant increase across all CQ dimensions. - **Control group:** Non-significant increase across all CQ dimensions.  \| **Group** \| **CQ Dimension** \| **Pre-M** \| **Post - M** \| **p-value** \| **Change** \| \| --- \| --- \| --- \| --- \| --- \| --- \| \| Experimental \| Metacognitive CQ \| 5.26 \| 5.77 \| 0.000 \| Significant \| \|  \| Cognitive CQ \| 3.88 \| 4.69 \| 0.000 \| Significant \| \|  \| Motivational CQ \| 5.72 \| 6.09 \| 0.000 \| Significant \| \|  \| Behavioral CQ \| 5.04 \| 5.70 \| 0.000 \| Significant \| \| Control \| Metacognitive CQ \| 5.11 \| 5.14 \| 0.294 \| Non-significant \| \|  \| Cognitive CQ \| 3.75 \| 3.86 \| 0.053 \| Non-significant \| \|  \| Motivational CQ \| 5.80 \| 5.74 \| 0.074 \| Non-significant \| \|  \| Behavioral CQ \| 5.05 \| 5.01 \| 0.224 \| Non-significant \|   **Note:** No data available for standard deviation scores. |
| 1. **Fakhreldin, Youssef and Anis, 2021** | Mixed delivery | Cultural Intelligence Scale (CQS) | Experimental group  (n= 108)  Control group  (n= 132) | - **Statistical analysis method**: Paired sample t-test. - **Experimental group:** Significant increase across all CQ dimensions. - **Control group:** Non-significant increase across all CQ dimensions.  \| **CQ Dimension**  **Experimental Group** \| **Mean Difference  (p-value)** \| **Change** \| \| --- \| --- \| --- \| \| Overall CQ \| p = .001 \| Significant \| \| Metacognitive CQ \| p = .002 \| Significant \| \| Cognitive CQ \| p = .000 \| Significant \| \| Motivational CQ \| p = .001 \| Significant \| \| Behavioral CQ \| p = .001 \| Significant \|   **Note:** No data available for mean and standard deviation scores. |
| 1. **Fischer, 2011** | Mixed delivery | Cultural Intelligence Scale (CQS) | Time 1:  (n=88)  Time 2:  (n= 68) | - **Statistical analysis method**: Mixed effects analysis of variance (ANOVA). - Significant decrease in cognitive, metacognitive CQ; non-significant decrease in motivational CQ, and non-significant increase in behavioral CQ from Time 1 to Time 2.  \| **CQ Dimension** \| **Time 1**  **(M ± SD)** \| **Time 2  (M ± SD)** \| **Change** \| **Effect size (d)** \| **Confidence Interval (CI 95%)** \| \| --- \| --- \| --- \| --- \| --- \| --- \| \| **Cognitive CQ** \| 3.85 **±** 1.30 \| 3.49 **±** 1.23 \| Decrease \| -0.28 \| [CI -0.601-0.035] \| \| **Metacognitive CQ** \| 4.82 **±** 1.35 \| 4.72 **±** 1.11 \| Decrease \| -0.08 \| [CI -0.397-0.237] \| \| **Motivational CQ** \| 5.36 **±** 1.08 \| 5.32 ± 0.97 \| Slight decrease \| -0.04 \| [CI -0.355-0.278] \| \| **Behavioral CQ** \| 5.14 **±** 1.37 \| 5.31 **±** 0.90 \| Increase \| 0.14 \| [CI -0.174-0.46] \| |
| 1. **Harris, McQuery, Raab and Elmore, 2008** | Didactic delivery | The Boston Survey of Culturally Competent Residency Training Practices in Psychiatry Questionnaire  & follow up questionnaire | Immediate follow-up:  Pre-post assessment (n=10)  9-month  follow- up:  Pre-post assessment (n=6) | - **Statistical analysis method:** Paired sample t-test. - Significant increase in overall cultural competence scores at the immediate follow-up. - Significant decrease in ‘Awareness of Privilege’ scores at the nine-month follow-up. All other scores showed non-significant changes from the immediate follow-up.  \| **Dimension** \| **Follow-Up** \| **Pre-M** \| **Post-M** \| **p-value** \| **Effect Size (d)** \| \| --- \| --- \| --- \| --- \| --- \| --- \| \| **Multicultural Knowledge and Skills** \| Immediate Follow-Up \| 2.90 \| 4.00 \| < 0.01 \| 0.58 \| \| **Awareness of Cultural Background** \| Immediate Follow-Up \| 2.90 \| 4.30 \| < 0.01 \| 0.91 \| \| **Awareness of Privilege** \| Immediate Follow-Up \| 2.90 \| 4.50 \| < 0.01 \| 0.98 \| \| **Clinical Application** \| Immediate Follow-Up \| 3.25 \| 3.74 \| < 0.01 \| 0.58 \| \| **Awareness of Privilege** \| Nine-Month Follow-Up \| 4.00 \| 4.67 \| < 0.05 \| 0.37 \|   **Note:** No data available standard deviation scores. |
| **13. Kirste and Holtbrügge, 2019** | Didactic delivery | Cultural Intelligence Scale (CQS) | Experimental group (n=32)  Control group  (n=18) | - **Statistical analysis method:** Wilcoxon signed rank test statistic (t0 to t1). - **Experimental group**: Non-significant changes in overall CQ scores or in any of the dimensions from time 0 to time 1. - **Control group**: Non-significant changes in overall CQ scores or in any of the dimensions from time 0 to time 1.  \| **Group** \| **CQ Dimension** \| **Z-value** \| **Asymptotic Significance** **(2-tailed)** \| **Effect Size (r)** \| \| --- \| --- \| --- \| --- \| --- \| \| **Experimental** \| Behavioral CQ \| -1.397 \| 0.162 \| -0.175 \| \|  \| Cognitive CQ \| -1.293 \| 0.196 \| -0.162 \| \|  \| Metacognitive CQ \| -1.138 \| 0.255 \| -0.142 \| \|  \| Motivational CQ \| -0.036 \| 0.971 \| -0.005 \| \|  \| Overall CQ \| -0.215 \| 0.830 \| -0.027 \| \| **Control** \| Behavioral CQ \| -0.311 \| 0.756 \| -0.052 \| \|  \| Cognitive CQ \| -1.040 \| 0.298 \| -0.173 \| \|  \| Metacognitive CQ \| -1.094 \| 0.274 \| -0.182 \| \|  \| Motivational CQ \| -1.200 \| 0.230 \| -0.200 \| \|  \| Overall CQ \| -1.089 \| 0.276 \| -0.182 \|   **Note:** Confidence intervals are not provided because the Wilcoxon signed-rank test is non-parametric, relying on ranks rather than means and standard deviations, and does not assume normality. |
| 1. **Kurpis and Hunter, 2017** | Mixed delivery | Cultural Intelligence Scale (CQS) | Domestic students (n=35)  International students (n=34) | - **Statistical analysis method:** Analysis of Variance (ANOVA). - International students significantly outperformed domestic students in cognitive CQ. - There were no significant differences between international and domestic students in metacognitive CQ, motivational CQ, or behavioral CQ, with similar scores across both groups.  \| **CQ Dimension** \| **International Students (M ± SD)** \| **Domestic Students (M ± SD)** \| **Change** \| **Effect size (d)** \| **Confidence Interval (CI 95%)** \| \| --- \| --- \| --- \| --- \| --- \| --- \| \| Cognitive CQ \| 4.85 ± 1.09 \| 4.02 ± 1.01 \| Significant \| -0.79 \| [CI -1.279 - 0.299] \| \| Metacognitive CQ \| 5.51 ± 0.89 \| 5.47 ± 0.96 \| Non- significant \| - 0.04 \| [CI -0.515 - 0.429] \| \| Motivational CQ \| 5.85 ± 0.82 \| 5.97 ± 0.76 \| Non- significant \| 0.15 \| [CI -0.321 - 0.624] \| \| Behavioral CQ \| 5.41 ± 0.73 \| 5.15 ± 0.87 \| Non-significant \| -0.32 \| [CI-0.799 - 0.151] \| |
| 1. **MacNab, 2012** | Experiential delivery | Cultural Intelligence Scale (CQS) | n=373 | - **Statistical analysis method:** Paired sample t-test. - Significant increase across all CQ dimensions, with cognitive CQ was not involved.  \| **CQ Dimension** \| **Pre-M** \| **Post-M** \| **t-test** \| **Percentage Increase** \| **p-value Significance** \| \| --- \| --- \| --- \| --- \| --- \| --- \| \| **Metacognitive CQ** \| 3.28 \| 4.27 \| 25.6 \| 30.3% \| < .001 Significant \| \| **Motivational CQ** \| 3.50 \| 4.07 \| 17.8 \| 16.5% \| < .001 Significant \| \| **Behavioral CQ** \| 3.35 \| 4.00 \| 22.5 \| 19.5% \| < .001 Significant \|   **Note:** No data available for standard deviation scores. |
| 1. **Majda, Zalewska-Puchała, Bodys-Cupak, Kurowska and Barzykowsk, 2021** | Experiential delivery | Cultural Intelligence Scale (CQS)  Cross-Cultural Competence Inventory (CCCI) | Study 1 (n=64)   Study 2 (n=66) | - **Statistical analysis method:** Paired sample t-test. - **Study 1:** Significant increase across all CQ dimensions but not in cross-cultural competence.  \| **CQ Dimension** \| **Pre- (M ± SD)** \| **Post-  (M± SD)** \| **p-value** \| **Effect Size (d)** \| **Confidence Interval  (CI 95%)** \| \| --- \| --- \| --- \| --- \| --- \| --- \| \| **CQ Overall** \| 76.77 **±** 19.20 \| 85.75 **±** 20.14 \| 0.001 \| 0.69 \| [CI -0.04-0.953] \| \| **Metacognitive CQ** \| 16.03 **±** 4.51 \| 18.19 **±** 4.82 \| 0.001 \| 0.55 \| [CI -0.034-0.959] \| \| **Cognitive CQ** \| 20.30 **±** 5.80 \| 23.00 **±** 6.06 \| 0.001 \| 0.53 \| [CI -0.041-0.952] \| \| **Motivational CQ** \| 20.47 **±** 6.61 \| 21.86 **±** 6.19 \| 0.001 \| 0.36 \| [CI -0.274-0.709] \| \| **Behavioral CQ** \| 19.97 **±** 6.70 \| 22.70 **±** 6.00 \| 0.001 \| 0.53 \| [CI -0.066-0.925] \|  \| **CCCI Dimension** \| **Pre- (M ± SD)** \| **Post-  (M ± SD)** \| **p-value** \| **Effect Size (d)** \| **Confidence Interval (CI 95%)** \| \| --- \| --- \| --- \| --- \| --- \| --- \| \| **CCCI Overall** \| 217.27 **±** 25.26 \| 220.51 **±** 22.73 \| 0.127 \| 0.14 \| [CI -0.356-0.625] \| \| **Cultural Adaptation** \| 77.02 **±** 11.48 \| 78.73 **±** 11.63 \| 0.081 \| 0.15 \| [CI -0.343-0.639] \| \| **Self-Presentation** \| 12.71 **±** 3.65 \| 13.16 **±** 4.29 \| 0.352 \| 0.11 \| [CI -0.377-0.603] \| \| **Ambiguity/Uncertainty Tolerance** \| 30.98 **±** 7.87 \| 31.20 **±** 7.47 \| 0.758 \| 0.03 \| [CI -0.461-0.519] \| \| **Determination** \| 23.78 **±** 4.81 \| 23.29 **±** 4.67 \| 0.353 \| -0.10 \| [CI -0.594-0.387] \| \| **Willingness to Engage** \| 42.20 **± 6.48** \| 43.42 **±** 5.99 \| 0.068 \| 0.19 \| [CI -0.303-0.679] \| \| **Mission** \| 30.58 **±** 4.10 \| 30.71 **±** 3.55 \| 0.785 \| 0.03 \| [CI -0.456-0.524] \|   **Study 2:** Significant increase in overall CQ across all dimensions but not in cross-cultural competence.   \| **CQ Dimension** \| **Pre-  (M ± SD)** \| **Post-M**  **(M ± SD)** \| **p-value** \| **Effect Size (d)** \| **Confidence Interval (CI 95%)** \| \| --- \| --- \| --- \| --- \| --- \| --- \| \| **CQ Overall** \| 79.62 **±** 18.39 \| 86.02 **±** 20.64 \| 0.010 \| 0.37 \| [CI -0.158-0.813] \| \| **Metacognitive CQ** \| 17.48 **±** 4.80 \| 18.41 **±** 4.23 \| 0.040 \| 0.23 \| [CI -0.278-0.689] \| \| **Cognitive CQ** \| 20.09 **±** 4.74 \| 22.17 **±** 6.59 \| 0.030 \| 0.34 \| [CI -0.124-0.849] \| \| **Motivational CQ** \| 21.15 **±** 6.43 \| 21.82 **±** 6.19 \| 0.050 \| 0.14 \| [CI -0.377-0.589] \| \| **Behavioral CQ** \| 20.89 **±** 6.78 \| 23.62 **±** 6.35 \| 0.020 \| 0.47 \| [CI -0.072-0.903] \|  \| **CCI Dimension** \| **Pre- (M ± SD)** \| **Post- (M ± SD)** \| **p-value** \| **Effect size (d)** \| **Confidence Interval (95% CI)** \| \| --- \| --- \| --- \| --- \| --- \| --- \| \| **CCCI Overall** \| 220.63 **±** 20.38 \| 221.02 **±** 21.53 \| 0.845 \| 0.02 \| [CI -0.464-0.501] \| \| **Cultural Adaptation** \| 79.13 **±** 10.11 \| 79.00 **±** 10.63 \| 0.904 \| -0.01 \| [CI -0.495-0.47] \| \| **Self-Presentation** \| 12.48 **±** 4.04 \| 12.75 **±** 3.79 \| 0.457 \| 0.07 \| [CI -0.414-0.552] \| \| **Ambiguity/Uncertainty Tolerance** \| 30.38 **±** 7.00 \| 29.13 **±** 6.86 \| 0.087 \| -0.18 \| [CI -0.664-0.303] \| \| **Determination** \| 22.69 **±** 4.88 \| 23.25 **±** 4.44 \| 0.304 \| 0.14 \| [CI -0.342-0.625] \| \| **Willingness to Engage** \| 44.08 **±** 6.40 \| 45.19 **±** 6.02 \| 0.084 \| 0.18 \| [CI -0.305-0.662] \| \| **Mission** \| 31.87 **±** 3.50 \| 31.69 **±** 4.24 \| 0.712 \| -0.05 \| [CI -0.529-0.436] \| |
| 1. **Presbitero and Toledano, 2018** | Mixed delivery | Cultural Intelligence Scale (CQS) | n=252 | - **Statistical analysis method**: Paired sample t-test. - Significant increase in overall CQ scores from pre- to post-intervention.  \| **CQ Domain** \| **Pre-  (M ± SD)** \| **Post-  (M ± SD)** \| **Effect Size (d)** \| **Confidence Interval (95% CI)** \| **p-value** \| \| --- \| --- \| --- \| --- \| --- \| --- \| \| Example CQ Domain \| 3.38 ± 1.77 \| 4.50 ± 1.44 \| 0.69 \| [CI 0.44-0.948] \| < 0.01 \| |
| 1. **Rahayu and Arga, 2019** | Experiential delivery | Observations with the indicators of cross-cultural competency | n=99 | - **Statistical analysis method:** Paired sample t-test. - Significant increase in cultural competence for the experimental group compared to the control group.  \| **Group** \| **Pre-M** \| **Post-M** \| **p-value** \| **Significance** \| \| --- \| --- \| --- \| --- \| --- \| \| Experimental \| 66 \| 78 \| 0.000 \| Significant \| \| Control \| 65 \| 71 \| 0.019 \| Significant \|   **Note:** No data available for standard deviation. |
| 1. **Ramsey and Lorenz, 2016** | Didactic delivery | Cultural Intelligence Scale (CQS) | - Experimental group (n=152) - Control group (n= 129) | - **Statistical analysis method**: Paired sample t-test. - **Experimental group**: Significant increase in overall CQ scores. - **Control group**: Non-significant increase in overall CQ scores.  \| **Group** \| **Pre-  (M ± SD)** \| **Post-  (M ± SD)** \| **p-value** \| **Effect Size (d)** \| \| --- \| --- \| --- \| --- \| --- \| \| Experimental \| 3.76 ± 0.76 \| 4.18 ± 0.69 \| < 0.001 \| 0.58 \| \| Control \| 3.54 ± 0.82 \| 3.61 ± 0.79 \| ns \| 0.09 \| |
| 1. **Rehg, Gundlach and Grigorian, 2012** | Didactic delivery | Cultural Intelligence Scale (CQS) | n=110 | - **Statistical analysis method**: Paired sample t-test. - **Class 1**: Significant increase in cognitive CQ, with positive but not significant changes in motivational and behavioral CQ. - **Class 2:** Significant increase in both cognitive and behavioral CQ, with a positive but not significant change in motivational CQ. - **Combined Results (Class 1 + Class 2):** Significant increase in both behavioral and cognitive CQ, with a positive but not significant increase in motivational CQ.   **Class 1:**   \| **CQ Dimension** \| **Pre**  **(M ± SD)** \| **Post  (M ± SD)** \| **Increase** \| **p-value** \| **Effect size (d)** \| **Confidence Interval  (95% CI)** \| \| --- \| --- \| --- \| --- \| --- \| --- \| --- \| \| Cognitive CQ \| 3.93 **±** 0.89 \| 4.90 **±** 0.91 \| 0.97 \| p < 0.01 \| 1.08 \| [CI 0.678-1.478] \| \| Motivational CQ \| 5.77 **±** 0.79 \| 5.85 **±** 0.80 \| 0.08 \| ns \| 0.10 \| [CI -0.273-0.475] \| \| Behavioral CQ \| 5.02 **±** 0.95 \| 5.22 **±** 0.62 \| 0.22 \| ns \| 0.25 \| [CI -0.126-0.625] \|   **Class 2:**   \| **CQ Dimension** \| **Pre  (M ± SD)** \| **Post  (M ± SD)** \| **Increase** \| **p-value** \| **Effect size (d)** \| **Confidence Interval  (95% CI)** \| \| --- \| --- \| --- \| --- \| --- \| --- \| --- \| \| Cognitive CQ \| 3.88 **±** 1.00 \| 4.57 **±** 0.98 \| 0.69 \| p <0.001 \| 0.70 \| [CI 0.312-1.082] \| \| Motivational CQ \| 5.35 **±** 1.07 \| 5.54 **±** 0.87 \| 0.19 \| ns \| 0.20 \| [CI -0.18-0.569] \| \| Behavioral CQ \| 4.90 **±** 0.94 \| 5.26 **±** 0.80 \| 0.36 \| p <0.01 \| 0.41 \| [CI 0.035-0.79] \|   **Class 1 + 2:**   \| **CQ Dimension** \| **Pre  (M ± SD)** \| **Post  (M ± SD)** \| **Increase** \| **p-value** \| **Effect size (d)** \| **Confidence Interval (CI 95%)** \| \| --- \| --- \| --- \| --- \| --- \| --- \| --- \| \| Cognitive CQ \| 3.89 **±** 0.96 \| 4.68 **±** 0.95 \| 0.79 \| p <0.001 \| 0.83 \| [CI 0.438-1.217] \| \| Motivational CQ \| 5.48 **±** 1.00 \| 5.64 **±** 0.84 \| 0.16 \| p <0.01 \| 0.17 \| [CI -0.201-0.548] \| \| Behavioral CQ \| 4.94 **±**0.93 \| 5.25 **±** 0.74 \| 0.31 \| p <0.01 \| 0.37 \| [CI -0.008-0.746] \| |
| 1. **Smith and Bahr, 2014** | Didactic delivery | Multicultural Awareness Knowledge Skills Survey  (MAKKS) | Pre-test  (n= 57)  Post-test  (n=26) | - **Statistical analysis method**: Paired sample t-test. - Significant increase in cultural competence across all MAKSS subscales, with effect sizes ranging from moderate to high.  \| **MAKSS Subscale** \| **Pre (M ± SD)** \| **Post (M ± SD)** \| **p-value** \| **Effect Size (d)** \| **Confidence Interval (95% CI)** \| \| --- \| --- \| --- \| --- \| --- \| --- \| \| **Cultural Awareness** \| 2.66 ± 0.19 \| 2.74 ± 0.16 \| < 0.01 \| 0.46 \| [ CI -0.027 - 0.91] \| \| **Cultural Knowledge** \| 2.62 ± 0.23 \| 2.80 ± 0.24 \| < 0.001 \| 0.76 \| [CI 0.294 - 1.25] \| \| **Cultural Skills** \| 2.73 ± 0.33 \| 2.94 ± 0.34 \| < 0.05 \| 0.63 \| [CI 0.157 - 1.104] \| |
| 1. **Wood and Peters, 2014** | Experiential delivery | Cultural Intelligence Scale (CQS) | n=42 | - **Statistical analysis method**: Paired sample t-test. - Significant increase in metacognitive, cognitive, and motivational CQ scores, with non-significant increase in behavioral CQ.  \| **CQ Dimension** \| **Time 1  (M ± SD)** \| **Time 2**  **(M ± SD)** \| **p-value** \| **Effect Size (d)** \| **Confidence Interval (95% CI** \| \| --- \| --- \| --- \| --- \| --- \| --- \| \| Metacognitive CQ \| 4.80 **±** 1.24 \| 5.64 **±** 0.72 \| 0.0003 \| 0.83 \| [CI 0.198-1.459] \| \| Cognitive CQ \| 3.36 **±** 1.18 \| 4.24 **±** 1.03 \| 0.0004 \| 0.80 \| [CI 0.166-1.423] \| \| Motivational CQ \| 5.12 **±** 1.16 \| 5.61 **±**0.90 \| 0.0340 \| 0.47 \| [CI -0.141-1.085] \| \| Behavioral CQ \| 4.70 **±** 1.28 \| 5.21 **±**1.11 \| 0.0530 \| 0.43 \| [CI -0.186-1.037] \| |
| 1. **Young, Haffejee and Corsun, 2018** | Mixed delivery | Cultural Intelligence Scale (CQS) | Experimental group  (n= 73)  Control group (n=95) | - **Statistical analysis method**: Multivariate analysis of variance (MANOVA). - **Experimental group:** Significant increase in metacognitive and behavioral CQ. - **Control group:** Non-significant changes across all CQ dimensions.  \| **CQ Dimension** \| **Mean Difference  Score** \| **F-value** \| **p-value** \| \| --- \| --- \| --- \| --- \| \| Cognitive CQ \| -0.675 \| 2.927 \| p = 0.09 \| \| Metacognitive CQ \| 0.546 \| 8.826 \| p = 0.00 \| \| Behavioral CQ \| 1.588 \| 12.652 \| p = 0.00 \| \| Motivational CQ \| -0.138 \| 2.033 \| p = 0.16 \|   **Note:** No data available for standard deviation. |
